# Integrated brain and plasma dual-channel metabolomics to explore the treatment effects of *Alpinia oxyphylla*Fructus on Alzheimer’s disease

**DOI:** 10.1371/journal.pone.0285401

**Published:** 2023-08-08

**Authors:** Zhi Sun, Yuanyuan Zhang, Mengya Zhang, Shengnan Zhou, Wenbo Cheng, Lianping Xue, Peipei Zhou, Xiaojing Li, Zhibo Zhang, Lihua Zuo

**Affiliations:** 1 Department of Pharmacy, The First Affiliated Hospital of Zhengzhou University, Zhengzhou, Henan Province, China; 2 Henan Engineering Research Center of Clinical Mass Spectrometry for Precision Medicine, Zhengzhou, Henan Province, China; 3 Suzhou Institute of Biomedical Engineering and Technology, Chinese Academy of Sciences, Suzhou, China; University of Waterloo, CANADA

## Abstract

*Alpinia oxyphylla* Fructus, called Yizhi in Chinese, is the dried fruit of Alpinia oxyphylla Miquel. It has been used in traditional Chinese medicine to treat dementia and memory defects of Alzheimer’s disease for many years. However, the underlying mechanism is still unclear. In this study, we used a rat Alzheimer’s disease model on intrahippocampal injection of aggregated Aβ_1–42_ to study the effects of *Alpinia oxyphylla* Fructus. A brain and plasma dual-channel metabolomics approach combined with multivariate statistical analysis was further performed to determine the effects of *Alpinia oxyphylla* Fructus on Alzheimer’s disease animals. As a result, in the Morris water maze test, *Alpinia oxyphylla* Fructus had a clear ability to ameliorate the impaired learning and memory of Alzheimer’s disease rats. 11 differential biomarkers were detected in AD rats’ brains. The compounds mainly included amino acids and phospholipids; after *Alpinia oxyphylla* Fructus administration, 9 regulated biomarkers were detected compared with the AD model group. In the plasma of AD rats, 29 differential biomarkers, primarily amino acids, phospholipids and fatty acids, were identified; After administration, 23 regulated biomarkers were detected. The metabolic pathways of regulated metabolites suggest that *Alpinia oxyphylla* Fructus ameliorates memory and learning deficits in AD rats principally by regulating amino acid metabolism, lipids metabolism, and energy metabolism. In conclusion, our results confirm and enhance our current understanding of the therapeutic effects of *Alpinia oxyphylla* Fructus on Alzheimer’s disease. Meanwhile, our work provides new insight into the potential intervention mechanism of *Alpinia oxyphylla* Fructus for Alzheimer’s disease treatment.

## Introduction

Alzheimer’s disease (AD) is a chronic neurodegenerative disease characterized by memory loss and cognitive impairment [1]. It has two distinct pathological features, including senile plaques caused by abnormal deposition of amyloid beta-peptide (Aβ) and neurofibrillary tangles (NFTs) composed of hyperphosphorylated tau [2]. The incidence of AD is rising every year due to the ageing population. It currently affects more than 50 million people worldwide and is expected to affect 150 million people by 2050 [3]. However, the pathogenesis of Alzheimer’s disease (AD) is complex and involves several types of neurodegenerative dysfunction. Western medicine still lacks specific drugs and treatment methods to address AD [4]. AD is still an irreversible and incurable neurological disease. Therefore, finding the drugs to slow or reverse the progression of AD is crucial.

Traditional Chinese medicine (TCM) has been used for thousands of years to treat dementia. As one of the “four famous south medicines” in China. *Alpinia oxyphylla* Fructus(AOF), a homology of medicine and food plants, is the dried fruit of *Alpinia oxyphylla* Miquel., which contains many kinds of chemical constituents, such as essential oils, sesquiterpenes, flavones, diarylheptanoids, glycosides and steroids. It is widely used for treating dementia in ancient China and also in modern Chinese medicine hospitals [5–7]. However, up to now, the current relevant research of *A*. *oxyphylla* treat on AD mainly focus on several single target points and pathway [8–10]. The overall mechanism of AOF in enhancing learning and memory ability had been less explored. Therefore, We used brain and plasma dual-channel metabolomics to investigate the therapeutic effects of AOF and the mechanism underlying its ameliorating effects on the pathogenesis of AD.

Metabolomics is characterized by involving the whole metabolic network rather than individual metabolites, which is highly consistent with the synergistic characteristics of TCM, making it particularly suitable for the study of the potential mechanism of TCM on disease [11]. In addition, metabolomics, as a systematic research method, could not only identify and confirm pharmacological effects and disease models but also directly responds to the changes in biochemical processes. Among the analytical techniques available for metabolomics studies, the Ultra Performance Liquid Chromatography-Quadrupole/Orbitrap Mass Spectrometry (UHPLC-Q-Orbitrap HRMS) technology is a convenient tool for high-throughput metabolomics profiling due to its higher sensitivity and resolution. In this study, an UHPLC-Q-Orbitrap HRMS-based metabolomic analysis was performed in plasma and brain of control and AD model rats. As well, the therapeutic effects of AOF and the mechanism underlying its ameliorating effects on the pathogenesis of AD were also investigated for the first time using metabolomic strategy.

## Materials and methods

### Drugs and chemicals

*Alpinia oxyphylla* Miquel was purchased from Henan Tongrentang Drug Co., Ltd. (Henan, China) and authenticated by Professor Hanbing Li of TCM Department, Henan University of Chinese Medicine.

Standards for MS/MS analysis of differential metabolites were purchased from Sigma-Aldrich Co., Ltd (St. Louis, MO, US) and J&K Chemical Ltd. (Beijing, China). Aβ1–42 was purchased from J&K Chemical Ltd. (Beijing, China). Rabbit anti-Aβ antibody was purchased from Wuhan Servicebio Technology CO, LTD (GB 11307, Wuhan, China). 2-Chloro-L Phenylalanine was purchased from J&K Chemical Ltd. (Beijing, China). Ketoprofen and HPLC grade formic acid were obtained from Sigma-Aldrich Co., Ltd (St. Louis, MO, US). HPLC grade methanol was purchased from Fisher Scientific (Fair Lawn, NJ, USA). Purified water was offered by Wahaha Co., Ltd. (Hangzhou, China).

### Drug extraction and preparation

The air-dried fruits of *Alpinia oxyphylla* Miquel (10 kg) were powdered and added into the round-bottom flask and then extracted three times for 2 h each time by 95% ethanol (1:10, w/v) at 55–60°C. After being gathered and separated by membrane filtration, the ethanol extract was concentrated to dryness with a rotary evaporator at 40°C. Finally, the extract was stored for further use (4°C). Take appropriate amount of extract and add castor oil in proportion (2 g:1 mL), stirring evenly, and then add proper amount of pure water to make the dosage concentration 80 mg/mL before use. The preparation of the extracts is done in our own laboratory.

### AD modeling and drug administration

The animal experiments conformed to the ethical use of animals of the National Institute of Health guidelines and were approved by the Animal Ethics Committee of the First Affiliated Hospital of Zhengzhou University. Male 2-month-old Sprague Dawley (SD) rats, which weighed 250–300 g, were purchased from experimental animal centre of Henan province (Zhengzhou, China). All rats had free access to food and water under standard conditions (temperature 20°C ± 2°C, relative humidity 55% ± 10%, 12:12 h light/dark cycle). Rats were adaptively fed for 1 week before the experiment. Aβ_1–42_ was dissolved and diluted in sterile physiological saline with a concentration of 1 mg/mL and incubated for 7 days at 37°C to form neurotoxic fibrils before injection.

**Fig 1:**
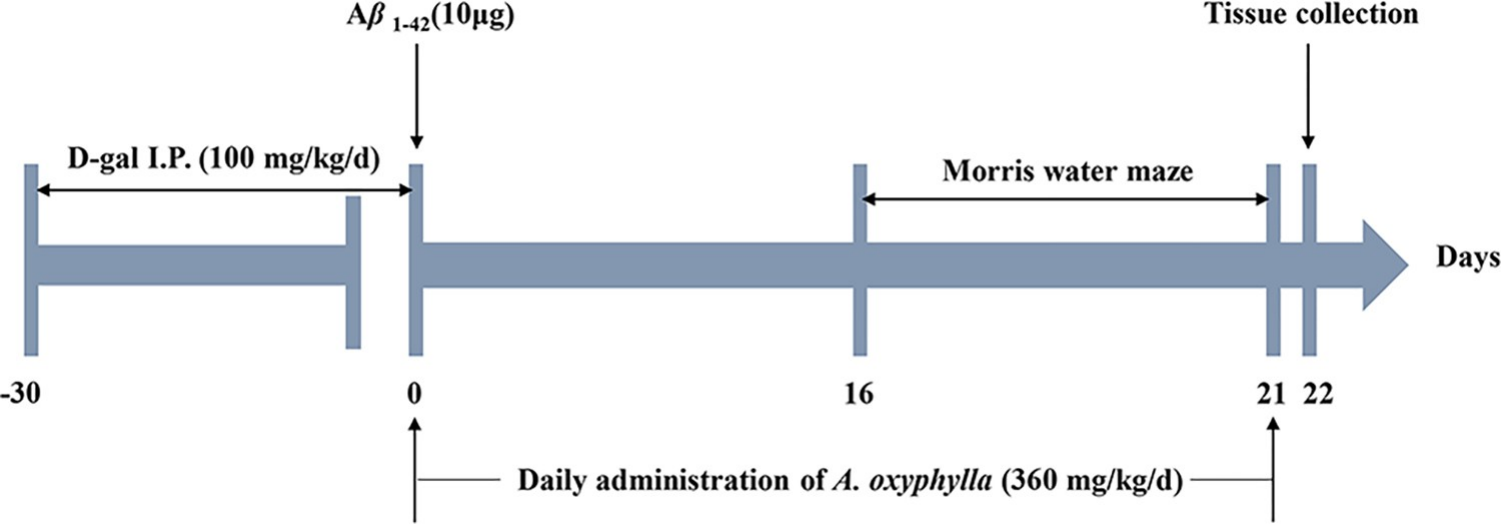
The experiment schedule.

After 1 week of adaptive feeding, all rats were randomly divided into 3 groups (n = 6/group): sham-operated group (S group: saline-lesioned, saline-treated), AD model group (M group: Aβ_1–42_ and D-gal-lesioned, saline-treated), and total extract group (T group: Aβ_1–42_ and D-gal-lesioned, treated with total extract of AOF). Administration of D-galactose aims to accelerate ageing in rats and ensure success in rat Alzheimer’s models. D-galactose (D-gal) was delivered by intraperitoneal injection once daily for 4 weeks in the model group and total extract group. The sham-operated group was injected with saline. The fifth week, rats were fasted for 12 h, then anesthetized and placed on a stereotaxic apparatus (RWD Inc., China). 10 μL of the aggregated A*β*1–42 peptide was injected into bilateral hippocampus region (AP, ± 3.5 mm, ML, ±2.0 mm, DV, -3.0 mm) according to stereotaxic atlas (The Rat Brain: In Stereotaxic Coordinates) The needle was removed with 5 min delay for diffusion. The sham-operated group was injected 10 μL of saline simultaneously. After surgery, penicillin sodium (i.m., 8 U/day) was administrated for 3 days for anti-inflammation therapy. Drug administration was performed after Aβ injection. Total extract of AOF (360 mg/kg/d) [12]was administered by gavage once daily for 3 weeks, while rats in sham-operated group and AD groups received normal saline of equivalent volume containing 8% castor oil. The whole procedure of experimental design is shown in **Fig 1**.

### Behavioral tests in MWM

After 2 weeks of administration, the Morris water maze (MWM) test was carried out to evaluate the spatial learning and reference memory ability [13]. The experimental device consists of a black circular pool (150 cm in diameter, 60 cm in height), a black circular platform (9 cm in diameter, 38.5 cm in height) and an image acquisition system. The circular pool was divided into four equal quadrants, and the target platform was placed at the midpoint of one quadrant. The camera was placed 2 m above the center of the bucket to record escape latencies and path length during each trial. Before the trial, water (25 ± 2°C) was poured into the pool and the water level was about 1.5 cm higher than the target platform. The trial included two parts: place navigation test and spatial probe test. Each rat was given two-place navigation test each day for five days. The rats were placed in the pool facing the wall and allowed to swim freely to seek target platform. If the rats failed to find the platform within 60 s, they need to be guided to platform and stayed for 10 s. In order to determine spatial learning ability of rats, space exploration test was performed on the sixth day. The test included removing the platform from the tank and allowing the rats to swim freely for 60 s. During the measurement process, the mouse movement was measured in both the target quadrant (in milliseconds)as well as the number of crossings(in times) (i.e., how many times the mouse crossed the area where the platform had previously been located). A computerized video imaging analysis system was used to record and analyze all data.

### Sample collection and preparation

After the MWM test, rats fasted overnight and blood samples were collected into heparinized EP tubes via the abdominal artery and centrifuged at 3500 rpm for 10 min and 4°C immediately. Then, the plasma was separated and stored at -80°C until metabolomics analysis. Finally, the skull was opened, and the brain was dissected out and put into normal saline solution to remove the blood and blotted on filter paper. Half of the brain tissue samples were frozen and stored at -80°C for further metabolomics analysis. The other half of the brain tissue was fixed in 4% paraformaldehyde and stored at 4°C for immunohistochemical staining.

After taking all samples from hypothermia refrigerator (-80°C) and thawing them on ice, 50 μL plasma of each sample was mixed to form the pooled plasma as the quality control (QC) sample. 300 μL methanol solution, containing 50 ng/mL L-2-chlorophenyl alanine and 500 ng/mL ketoprofen as the internal standard, was added to 100 μL the plasma (including QC sample) sample to precipitate protein and vortexed for 3 min. After centrifuging at 13,000 rpm, 4°C for 15 min, a 5 μL aliquot of the solution was injected into the UHPLC-MS/MS for analysis.

The right half of the brain (200–300 mg) was thawed at 4°C, weighed and recorded. After adding 2 mL/g of 0.9% NaCl, brain tissues were homogenized by using a tissue lyser (TL2010S, DHS Co., LTD, Beijing). All the procedures were performed on ice. After vortex-mixing, 50 μL brain of each sample was mixed to form the pooled brain as the quality control (QC) sample, and a 100 μL aliquot of the brain homogenate was used for the metabolomics study. The next pretreatment method was the same as the plasma method.

### Immunohistochemistry

The histopathological changes of brain in rats were estimated by immunohistochemistry [14]. The brains, which had been fixed in 4% paraformaldehyde, were embedded in paraffin and sectioned at a thickness of 4 μm. After deparaffinization in xylene and rehydration in graded ethanol solutions, the sections were boiled in Citric acid (PH 6.0) Antigen Retrieval Solution (Servicebio technology CO., Wuhan, China) for 8 min for antigen retrieval. Endogenous peroxidase inactivation was performed by incubating sections in 3% hydrogen peroxide at 37°C for 25 min and blocking with rabbit serum for 30 min. The sections were incubated with rabbit anti-Aβ antibody (Servicebio technology CO., Wuhan, China) diluted at 1:500 at 4°C overnight. Sections were washed with 0.01 M phosphate-buffered saline, then incubated with HRP-labeled goat anti-rabbit IgG (1:500; Servicebio technology CO., Wuhan, China) at room temperature for 50 min. After washing, DAB Developer was added to the sections, and the degree of color rendering was observed under the microscope. The positive color was brown-yellow. Sections were washed with distilled water before counterstaining with hematoxylin and then dehydrated in a graded alcohol series, cleared in xylene. Images were observed and taken photographs under microscope.

### UHPLC-MS/MS system conditions

We used Q Exactive high-resolution mass spectrometry combined with an Ultra-High-Performance Liquid Chromatography (UHPLC) system was used to separate and identify the metabolites. Chromatographic separation was achieved on an ACQUITY UPLC® BEH C18 (2.1 × 100 mm, 1.7 μm, Waters, USA). The column temperature was set at 40°C, while the injection volume was 5 μL. Chromatographic analysis was accomplished with a gradient elution consisting of 0.1% formic acid in water (solvent A) and acetonitrile (solvent B) at a flow rate of 0.30 mL/min. The gradient elution was as follows: 0∼0.5 min, 95% A; 0.5∼8.0 min, 95%∼0% A; 8.0∼10.0 min, 0% A; 7.0∼9.0 min, 80∼100% A; 9.0∼11.0 min, 100% A; 11.0∼13.0 min, 5% A.

The mass spectrometer with an electrospray ionization (ESI) source was performed in the positive and negative ion mode. In the positive and negative ion mode, the spray voltage was set to + 3.5 kV and—2.8 kV, and the sheath gas flow rate was set to 40 μL/min and 38 μL/min, respectively. Data was acquired by Full MS/dd MS2 scan patterns using a scan range of 80 to 1200 m/z in the positive and negative mode. In full MS mode, the resolution was 70000. Moreover, the isolation window was set as 1.0 m/z with a resolution of 17,500 in dd MS2 mode. The gradient collision energy was at 20, 40, and 60 eV. The temperature of capillary was 320°C. The auxiliary gas was at a flow rate of 30 μL/min.

### Data preprocessing and identification of potential biomarker

QC samples were inserted at regular intervals (every five samples) throughout the entire experiment to verify the stability and reliability of metabolic profiling platform. The raw data of plasma and brain samples was imported into Compound Discoverer (CD) (Version 3.0, Thermo Scientific) for peak detection, alignment, normalization and other data pre-processing. Output three-dimensional data, consisting of sample name, spectral peak information (including retention time and molecular weight), and peak area, was imported into the SIMCA (version 14.0, Umetrics, Umea, Sweden) for multivariate statistical analysis containing principal component analysis (PCA) and orthogonal partial least square discriminant analysis (OPLS-DA). After brain and plasma samples were subjected to OPLS-DA for the screening of discriminant metabolites, permutation test was performed 200 times to assess whether the OPLS-DA models were over-fitting [15]. Variable Importance in Projection (VIP) value of metabolites was obtained from OPLS-DA model to directly find the potential metabolites. In order to further screen the potential metabolites between different groups, the student’s t-test and fold change value of all the detected peaks metabolites were carried out using MetaboAnalyst 4.0 online (http://www.metaboanalyst.ca). The potential biomarkers were identified by searching the Human Metabolome Database (HMDB) database (http://www.hmdb.ca) and MassBank of North America (MoNA) database (http://www.massbank.ca) and comparing their retention times, mass values, and MS/MS fragmentation. In addition, metabolic pathway analysis and heatmap analysis were generated with these screened metabolites by MetaboAnalyst 4.0 online.

### Statistical analysis

The spectra were Metabolomics-Based Research on Synergistic Effect selected from input LC-MS data files and retention time alignment was accomplished based on mass tolerance and time shift criteria. Preliminary identification of metabolites was realized by searching databases including HMDB, ChemSpider, Mass Lists, mzCloud, mzVault, and local database. Combining multiple nodes such as retention time comparison, unknown compound detection, unknown compound clustering, database search, blank filling, and region normalization, some of potential biomarkers identified with reference standards, an undirected metabolomics workflow for raw data processing is formed.

GraphPad Prism software (Version 9.4) was used in this study. All results are expressed as the means ± standard deviations. Repeated measures two-way ANOVA followed by post-hoc Tukey’s test was performed for the Morris water maze tests of the latency. Other group comparison was performed using one-way ANOVA. In all the tests, p < 0.05 was considered statistically significant.

## Results

### Behavioral tests in MWM

**Fig 2:**
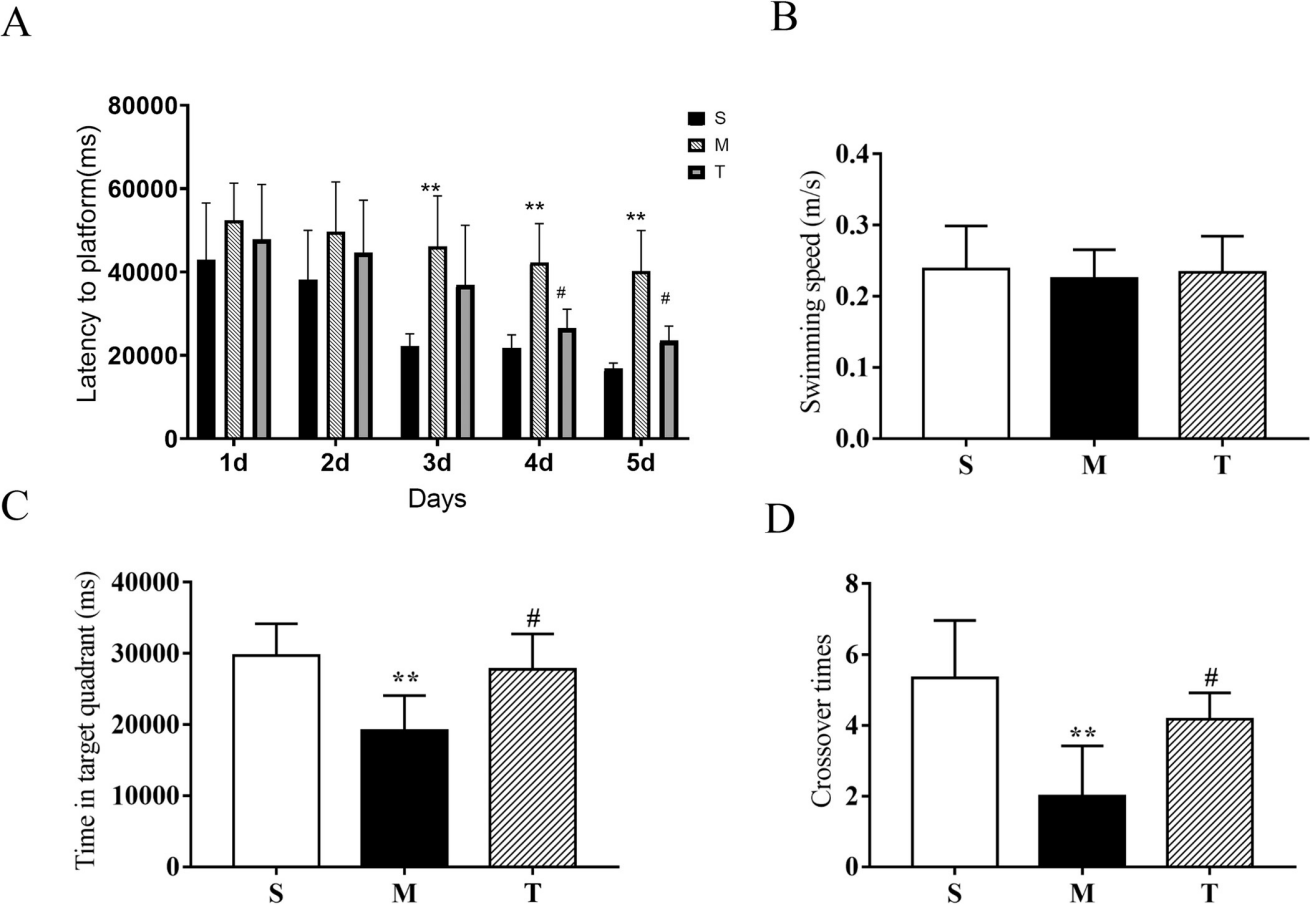
Effect of AOF on AD model rats in MWM. **(A)** Escape latency required for the rats to find the hidden platform over 5 consecutive training days; **(B)** Average swimming speeds of the rats during 5 training days; **(C)** Time in target quadrant in space exploration test (%). **(D)** Crossover times in previous platform location in space exploration test. Each column represents the mean ± SD for each group (n = 6 per group, * P < 0.05 versus sham group, ** P < 0.01 versus sham group; # P < 0.05 versus AD model group, ## P < 0.01 versus AD model group). S, sham group; M, AD model group; T, AOF group.

The performances of the rats in the Morris water maze (MWM) were shown in Fig 2. We had found that the escape latencies of rats in all groups were gradually decreased as the training proceed **(Fig 2A)**. From the third day, the escape latencies of the model group were significantly longer than that of sham group (P < 0.01). Furthermore, from the fourth day, compared with the AD model group, the cognition abilities of rats in the total extract group were elevated to some extent (P < 0.05). **Fig 2B** showed that there was no significant difference (P > 0.05) in swimming speed among all groups, which excluded the effect of the exercise capacity on experimental results. S1 Fig illustrated the swim paths of rats in the second trial of the second and fifth day in this test. Rats tended to explore all four quadrants of the pool in the second day. Since then, their path length had changed due to different memory levels. In the spatial probe test, the lengths of time in the target quadrant were markedly reduced **(Fig 2C)** and the number of crossing the platform was obviously decreased **(Fig 2D)** in the AD group compared to sham group (P < 0.01), while the AD rats treated with total extract of AOF showed a reverse trend (P < 0.05). These results indicated that AOF could effectively alleviate cognitive impairment in AD rats.

### Immunohistochemical results

**Fig 3:**
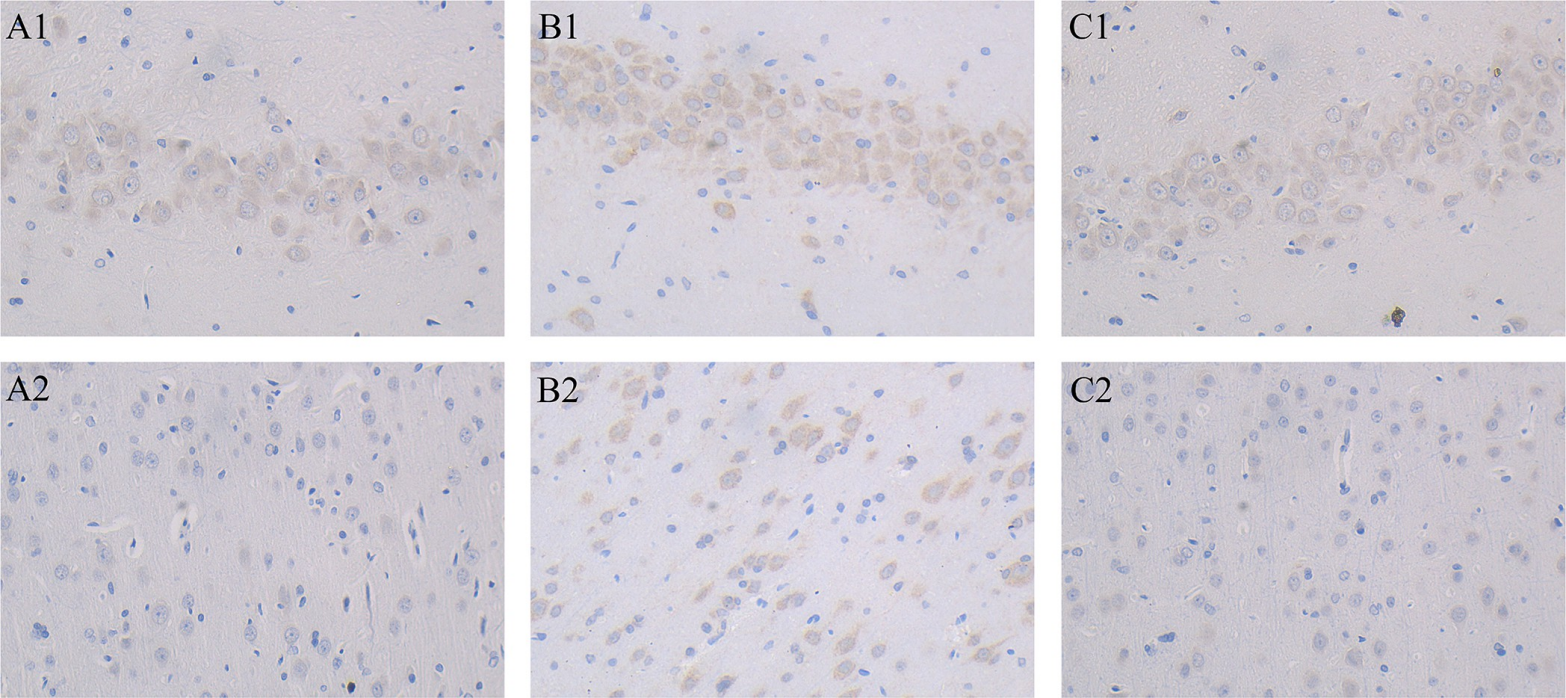
Aβ_1–42_ immunohistochemical staining in rat brain hippocampus. (×400); **A1:** sham group; **B1**: AD model group; **C1:** AOF group. Aβ_1–42_ immunohistochemical staining in rat brain cortex. (×400); **A2:** sham group; **B2:** AD model group; **C2:** AOF group. A statistical method was used for comparing the expression levels of Aβ in the hippocampi and cerebral cortex. The results showed that, compared with the sham group, the expression levels of Aβ deposition increased significantly in the AD group (P < 0.05). However, AOF could obviously reduce the expression levels of Aβ deposition (P < 0.05) (S2 Fig).

The different groups were observed and compared the changes in the neurons of the hippocampi of rats by Immunohistochemical staining. In Sham operation group, neurons had large nuclei with clear nucleoli, and a little light brown Aβ deposition was observed **(Fig 3A1)**. On the contrary, morphological changes were clearly observed: the neurons, with darkly stained nuclei, were surrounded by brown-yellow, scattered Aβ deposits **(Fig 3B1)**. In total extract group, the nucleoli were clearly visible and the Aβ deposits were light brown **(Fig 3C1)**. Meanwhile, pathological changes similar to those in the hippocampi were observed in the cerebral cortex. In the sham group, cortical cells had a regular arrangement, normal morphology and no Aβ deposition **(Fig 3A2)**. However, Sparse and degeneration of cells and surrounding brown-yellow depositions of Aβ can be found in the AD model group **(Fig 3B2)**. In the total extract group, there were more spares and light brown Aβ deposits compared to the AD group **(Fig 3C2)**. These results demonstrated that Aβ accumulation and neurodegeneration were ameliorated by total extract treatment.

### Metabolite profiling analysis

**Fig 4:**
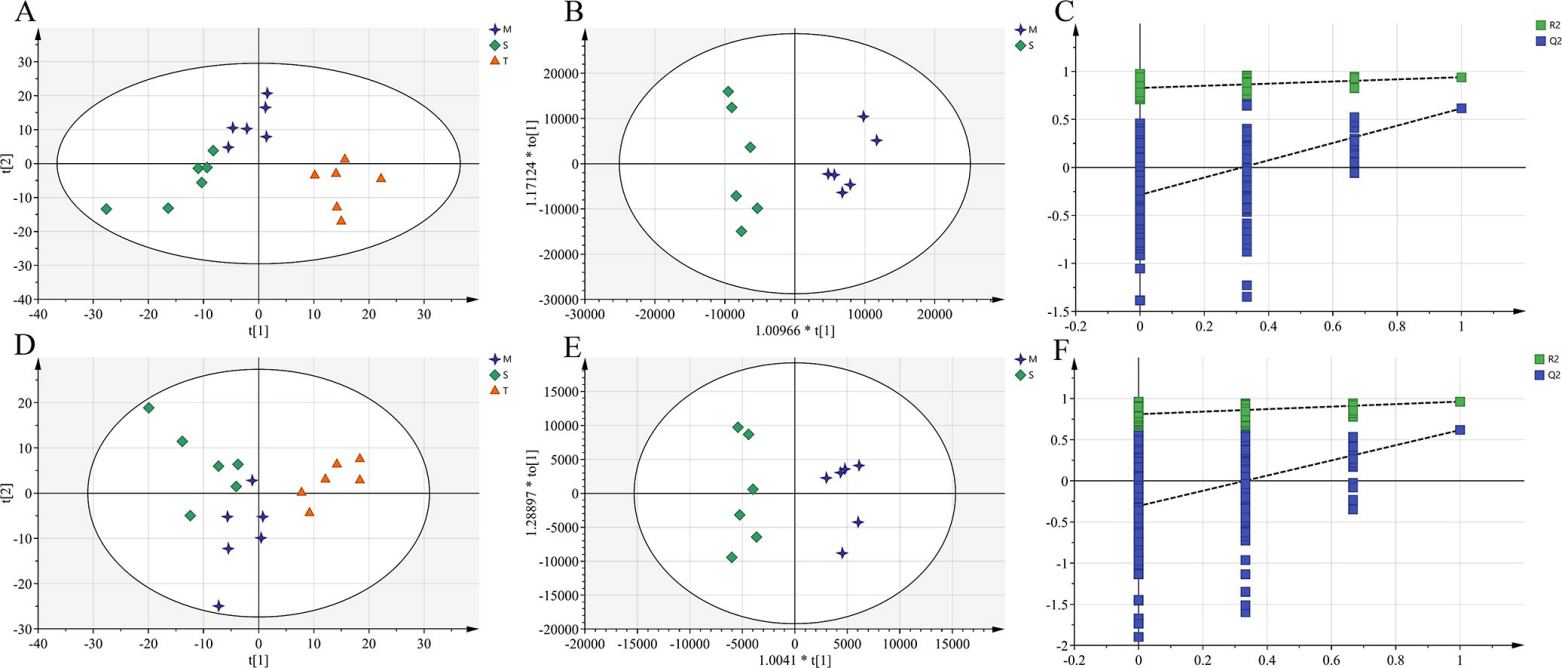
Multivariate data analyses of brain samples. PCA score plots of different groups in positive **(A)** and negative **(D)** ion mode; The OPLS-DA score plots from M group and S group in positive **(B)** and negative **(E)** ion mode; The permutations test of M vs. S group in positive **(C)** and negative **(F)** ion mode. S, sham group; M, AD model group; T, AOF group.

**Fig 5:**
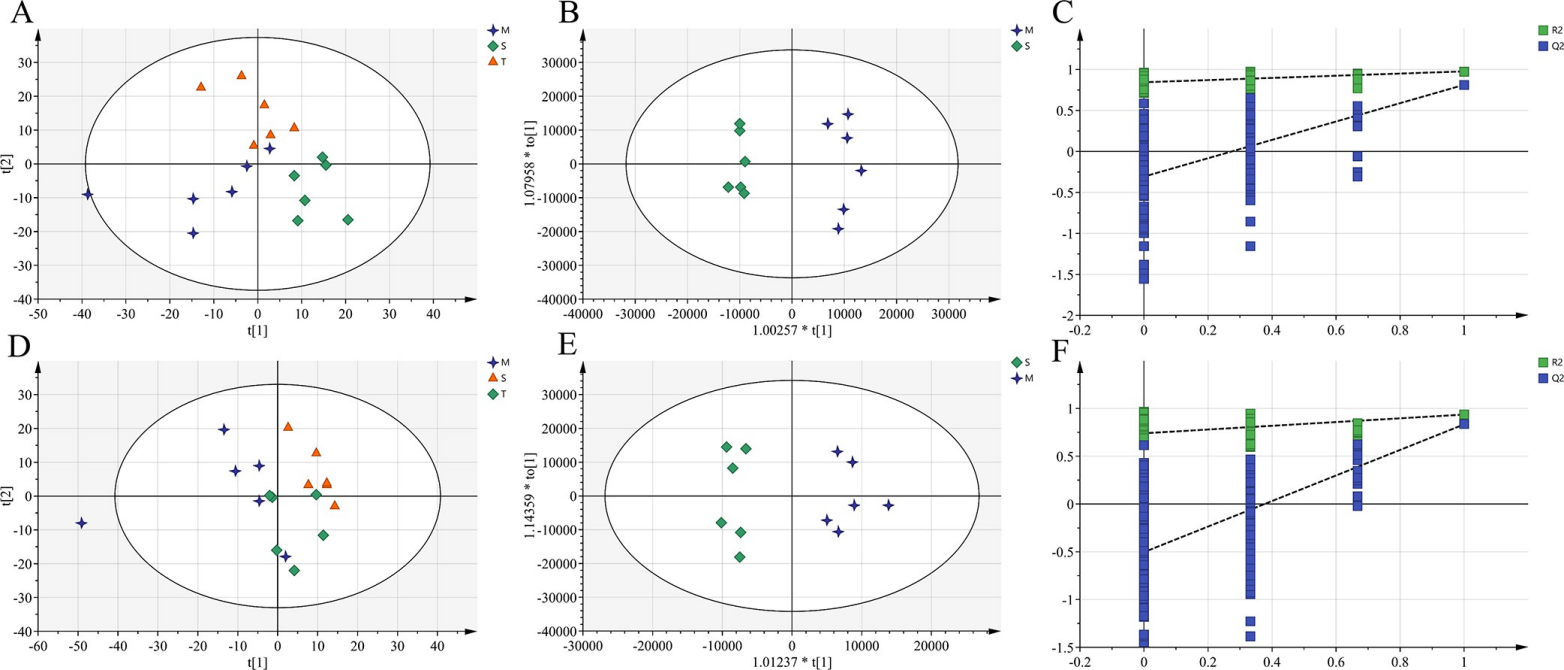
Multivariate data analyses of plasma samples. PCA score plots of different groups in positive **(A)** and negative **(D)** ion mode; The OPLS-DA score plots from M group and S group in positive **(B)** and negative **(E)** ion mode; The permutations test of M vs. S group in positive **(D)** and negative **(F)** ion mode. S, sham group; M, AD model group; T, AOF group.

Representative total ion chromatograms of brain and plasma samples obtained in both positive and negative ion modes are presented in **S3(A)–S3(C)** and **S4(A)–S4(C) Figs**, respectively. Although some changes in metabolites could be observed from chromatograms, only pattern recognition method could provide clear and detailed results. The data processed by CD software was submitted to the SIMCA software for multivariate statistical analysis. The close cluster of QC samples in the PCA scores plot showed good stability and repeatability of the metabolic profiling method **(S5 Fig)**. In order to investigate the global metabolism variations, PCA was first used to analyze all observations obtained in both ion modes. As shown in the PCA score plots **(Figs 4A, 4D**, **5A** and **5D**), an overview of all brain and plasma samples in the data was observed and showed a significant grouping trend between the sham operation group, the model group and the total extract group. This observation indicates that endogenous metabolites were changed in model group and total extract group.

### Identification of differential biomarkers in AD rats

The OPLS-DA model was established to further screen out differential metabolites in brain and plasma, and the separation between M and S group was very clearly presented in the scatter plot of the model (shown in **Figs 4B, 4E**, **5B** and **5E**). A permutation of 200 times was performed, and the result indicated that all the OPLS-DA models had good stability and reliability without over-fitting phenomenon in **Figs 4C, 4F**, **5C** and **5F**. The variable influence on project (VIP) value was obtained from OPLS-DA model to directly find the potential metabolites. Fold change values and Student’s t-test were also calculated to ensure that the detected metabolites had obvious concentration changes and statistically significant. Those metabolites with VIP > 1.0 and P < 0.05 were selected as potential biomarkers between different groups. Finally, 11 brain biomarkers and 29 plasma biomarkers were identified respectively, which contributed to the classification of the sham and model groups (summarized in **S1, S2 Tables**)

### Identification of regulated biomarkers influenced by AOF

As with the above method, the OPLS-DA model was established between M and T group. **S6A, S6C**, **S7A** and **S7C** **Figs** also showed a good separation. The results of permutation indicated the models without over-fitting (shown in S6B, S6D, S7B and S7D Figs). VIP value was obtained, and fold change values and Student’s t-test was also calculated. Finally, those metabolites with VIP > 1.0 and P < 0.05 were selected as potential biomarkers between different groups. In the result, 9 brain biomarkers and 23 plasma biomarkers were identified respectively, which were regulated by AOF (summarized in **S3, S4 Tables**).

**Fig 6:**
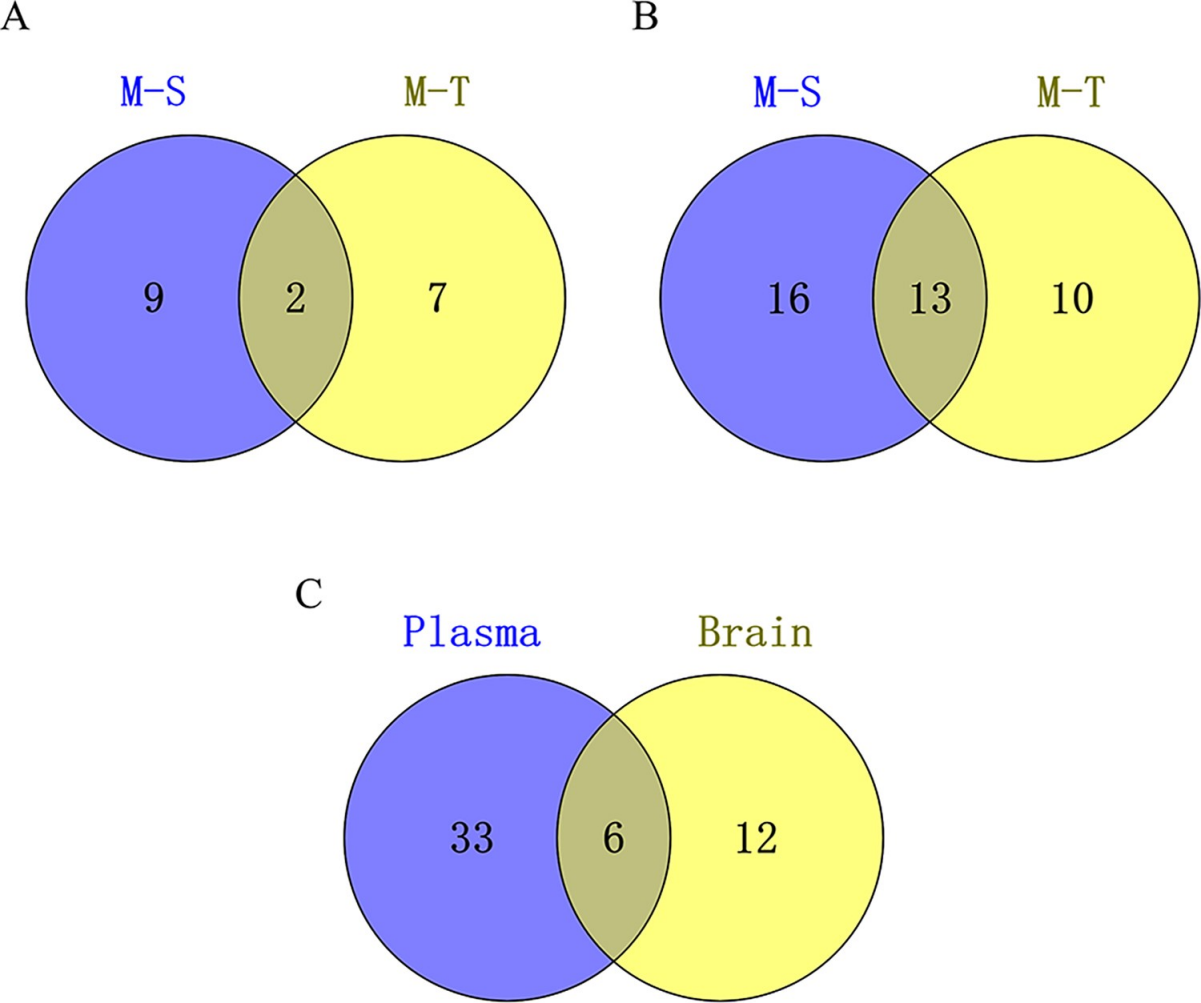
Venn diagrams illustrating overlap of differential biomarkers (M-S) and regulated biomarkers (M-T) in the brain **(A)** and plasma **(B)**. Venn diagrams illustrating overlap of the brain biomarkers and plasma biomarkers **(C)**. S, sham group; M, AD model group; T, AOF group.

By observing differential biomarkers and regulated biomarkers, we found that among the brain differential biomarkers, 2 biomarkers recovered significantly after administration; among the plasma differential biomarkers, 13 biomarkers also recovered significantly after administration. The relationship of biomarkers in different groups was demonstrated by Venn diagrams **(Fig 6A–6C)**. All identified brain and plasma biomarkers including regulated and differentiated biomarkers are shown in **Tables 1** and **2**, respectively.

**Table 1:** Differential biomarkers and regulated biomarkers identified in brain.

NO	RT (min)	M/Z	Adduct	Δppm	Metabolites	Formula	Fold change (M/S)	Adjusted by AOF Fold change (M/T)	Pathway involved
1	0.84	130.04964	[M+H]+	-1.766	Spermidine	C6H11NO2	0.77*	0.99	Amino acid metabolism
2	1.04	116.07036	[M+H]+	-2.112	(2R-3S)-2,3-Dimethylmalate	C5H9NO2	0.71#	0.97	Amino acid metabolism
3	1.07	130.04962	[M+H]+	-1.074	2-Hydroxy-2,4-pentadienoate	C5H7NO3	0.77	0.83*	Amino acid metabolism
4	1.08	118.08600	[M+H]+	-2.161	Succinic Semialdehyde	C5H11NO2	0.65#	0.92	Amino acid metabolism
5	1.10	175.11896	[M+H]+	0.044	Proline^R^	C6H14N4O2	0.74#	0.83*	Amino acid metabolism
6	1.10	150.05803	[M+H]+	-1.972	Pipecolic acid	C5H11NO2S	0.72#	0.92	Amino acid metabolism
7	1.12	145.06136	[M-H]-	-3.485	Choline	C5H10N2O3	0.79	0.85*	Nucleotide metabolism
8	1.23	204.12259	[M+H]+	-2.178	Carnitine	C9H17NO4	0.84	0.62*	Energy metabolism
9	1.34	191.01935	[M-H]-	-1.967	Trigonelline	C6H8O7	1.05	1.32*	Energy metabolism
10	1.35	165.05428	[M+H]+	-2.064	Glutamine^R^	C9H8O3	0.72#	0.94	Amino acid metabolism
11	1.58	132.10179	[M+H]+	-0.872	Crotonic acid	C6H13NO2	0.71#	0.99	Amino acid metabolism
12	3.50	188.07016	[M+H]+	-2.367	Isoleucine^R^	C11H9NO2	0.77#	0.97	Amino acid metabolism
13	8.34	524.27740	[M-H]-	0.447	Tryptophan^R^	C27H44NO7P	0.84*	0.84*	Glycerophospholipid metabolism
14	8.63	496.33804	[M+H]+	-3.477	3-Indoleacrylic acid	C24H50NO7P	0.95	0.85*	Glycerophospholipid metabolism
15	8.79	526.29346	[M-H]-	-0.860	Gamma-Glu-Leu	C27H46NO7P	0.73	0.56#	Glycerophospholipid metabolism
16	8.83	452.27805	[M-H]-	1.955	Hippuric acid	C21H44NO7P	0.87*	0.90	Glycerophospholipid metabolism
17	9.05	528.30956	[M-H]-	2.664	Phenyllactic acid	C27H48NO7P	0.85	0.75*	Glycerophospholipid metabolism
18	10.04	147.09138	[M+H]+	-2.005	Sphingosine	C9H10N2	1.39#	1.30	Metabolism of cofactors and vitamins

^R^represents potential biomarkers identified with reference standards.

RT: retention time.

LysoPC: lysophosphatidylcholine.

*The values have statistical significance (P < 0.05).

#The values have statistical significance (P < 0.01).

**Table 2:** Differential biomarkers and regulated biomarkers identified in plasma.

NO	RT (min)	M/Z	Adduct	Δppm	Metabolites	Formula	Fold change (M/S)		Adjusted by AOF Fold change (M/T)		Pathway involved
1	0.82	146.16475	[M+H]+	-2.902	Spermidine	C7H19N3	1.33		1.32*		Amino acid metabolism
2	0.99	161.04559	[M-H]-	0.269	(2R-3S)-2,3-Dimethylmalate	C6H10O5	0.62#		0.66#		Metabolism of cofactors and vitamins
3	0.99	113.02449	[M-H]-	0.643	2-Hydroxy-2,4-pentadienoate	C5H6O3	0.62#		0.66*		Amino acid metabolism
4	1.00	101.02435	[M-H]-	10.190	Succinic Semialdehyde	C4H6O3	0.63#		0.68*		Amino acid metabolism
5	1.00	116.07023	[M+H]+	-3.232	Proline^R^	C5H9NO2	0.76#		0.94		Amino acid metabolism
6	1.03	130.08594	[M+H]+	-2.653	Pipecolic acid	C6H11NO2	1.53*		1.11		Amino acid metabolism
7	1.05	104.10669	[M+H]+	-2.888	Choline	C5H13NO	1.02		2.01#		Glycerophospholipid metabolism
8	1.07	162.11201	[M+H]+	-2.837	Carnitine	C7H15NO3	0.66#		0.87		Energy metabolism
9	1.13	138.05466	[M+H]+	-2.137	Trigonelline	C7H7NO2	0.15#		0.57		Metabolism of cofactors and vitamins
10	1.19	145.06180	[M-H]-	7.110	Glutamine^R^	C5H10N2O3	1.49#		1.26#		Amino acid metabolism
11	1.46	87.04388	[M+H]+	-2.022	Crotonic acid	C4H6O2	3.40#		1.39		Lipid metabolism
12	1.54	132.10172	[M+H]+	-1.402	Isoleucine^R^	C6H13NO2	1.26*		1.25*		Amino acid metabolism
13	3.38	205.09705	[M+H]+	-0.508	Tryptophan^R^	C11H12N2O2	1.38#		1.23		Amino acid metabolism
14	3.46	188.07018	[M+H]+	-2.260	3-Indoleacrylic acid	C11H9NO2	1.36*		1.25		
15	3.63	261.14450	[M+H]+	0.007	Gamma-Glu-Leu	C11H20N2O5	1.63#		1.43*		Amino acid metabolism
16	4.06	180.06494	[M+H]+	-3.219	Hippuric acid	C9H9NO3	0.34#		0.79		Amino acid metabolism
17	4.72	165.05602	[M-H]-	8.478	Phenyllactic acid	C9H10O3	0.22#		0.45		Fatty acid metabolism
18	7.46	300.29138	[M+H]+	5.575	Sphingosine	C18H37NO2	1.03		1.87*		Lipid metabolism
19	7.60	313.23871	[M-H]-	4.386	12,13-Dihome	C18H34O4	5.21#		2.61*		Fatty acid metabolism
20	7.87	424.34094	[M+H]+	-2.817	Linoleoylcarnitine	C25H45NO4	1.75*		1.63		Energy metabolism
21	7.98	391.28558	[M-H]-	0.504	Deoxycholic acid	C24H40O4	2.14*		3.08#		Secondary bile acid biosynthesis
22	8.12	400.33914	[M+H]+	-7.482	Palmitoylcarnitine	C23H45NO4	1.84#		1.75#		Energy metabolism
23	8.19	426.35632	[M+H]+	-3.437	Oleoylcarnitine	C25H47NO4	1.76#		1.44*		Energy metabolism
24	8.38	544.33862	[M+H]+	-2.105	LysoPC(20:4)	C28H50NO7P	1.23*	1.20*		Glycerophospholipid metabolism		
25	8.40	520.33862	[M+H]+	-2.202	LysoPC(18:2)	C26H50NO7P	0.65#	0.95		Glycerophospholipid metabolism		
26	8.41	504.30978	[M-H]-	2.606	LysoPE(20:2)	C25H48NO7P	0.70#	0.91		Glycerophospholipid metabolism		
27	8.55	428.37231	[M+H]+	-2.627	Stearoylcarnitine	C25H49NO4	2.20#	1.18		Energy metabolism		
28	8.80	496.33850	[M+H]+	-2.550	1-Palmitoylglycerophosphocholine	C24H50NO7P	1.02	1.15*		Glycerophospholipid metabolism		
29	9.25	510.35379	[M+H]+	-3.186	LysoPC(17:0)	C25H52NO7P	1.00	1.48#		Glycerophospholipid metabolism		
30	9.68	301.21661	[M-H]-	1.339	Eicosapentaenoic acid^R^	C20H30O2	2.05#	3.67#		Fatty acid metabolism		
31	9.79	550.38574	[M+H]+	-1.773	PC(18:1(9Z)e/2:0)	C28H56NO7P	1.10	1.73#		Glycerophospholipid metabolism		
32	9.97	327.23306	[M-H]-	0.325	Docosahexaenoic acid^R^	C22H32O2	1.39	2.18#		Fatty acid metabolism		
33	10.11	303.23306	[M-H]-	3.968	Arachidonic acid^R^	C20H32O2	1.53#	1.73#		Fatty acid metabolism		
34	10.15	271.22797	[M-H]-	4.419	16-Hydroxyhexadecanoic acid	C16H32O3	1.79#	1.87*		Fatty acid metabolism		
35	10.32	279.23291	[M-H]-	3.772	Linoleic acid^R^	C18H32O2	1.42*	1.57		Fatty acid metabolism		
36	10.35	329.24881	[M-H]-	0.627	Docosapentaenoic acid(22n-6)^R^	C22H34O2	1.25	1.65*		Fatty acid metabolism		
37	10.59	331.26447	[M-H]-	0.653	Adrenic acid	C22H36O2	1.02	1.77#		Fatty acid metabolism		
38	10.75	281.24863	[M-H]-	3.994	Oleic acid^R^	C18H34O2	1.46#	1.32		Fatty acid metabolism		
39	11.48	283.26425	[M-H]-	3.859	Stearic acid^R^	C18H36O2	1.63*	1.12		Fatty acid metabolism		

^R^represents potential biomarkers identified with reference standards.

RT: retention time.

LysoPC: lysophosphatidylcholine.

LysoPE: lysophosphatidylethanolamine.

*The values have statistical significance (P < 0.05).

#The values have statistical significance (P < 0.01).

### Metabolite data analysis

**Fig 7:**
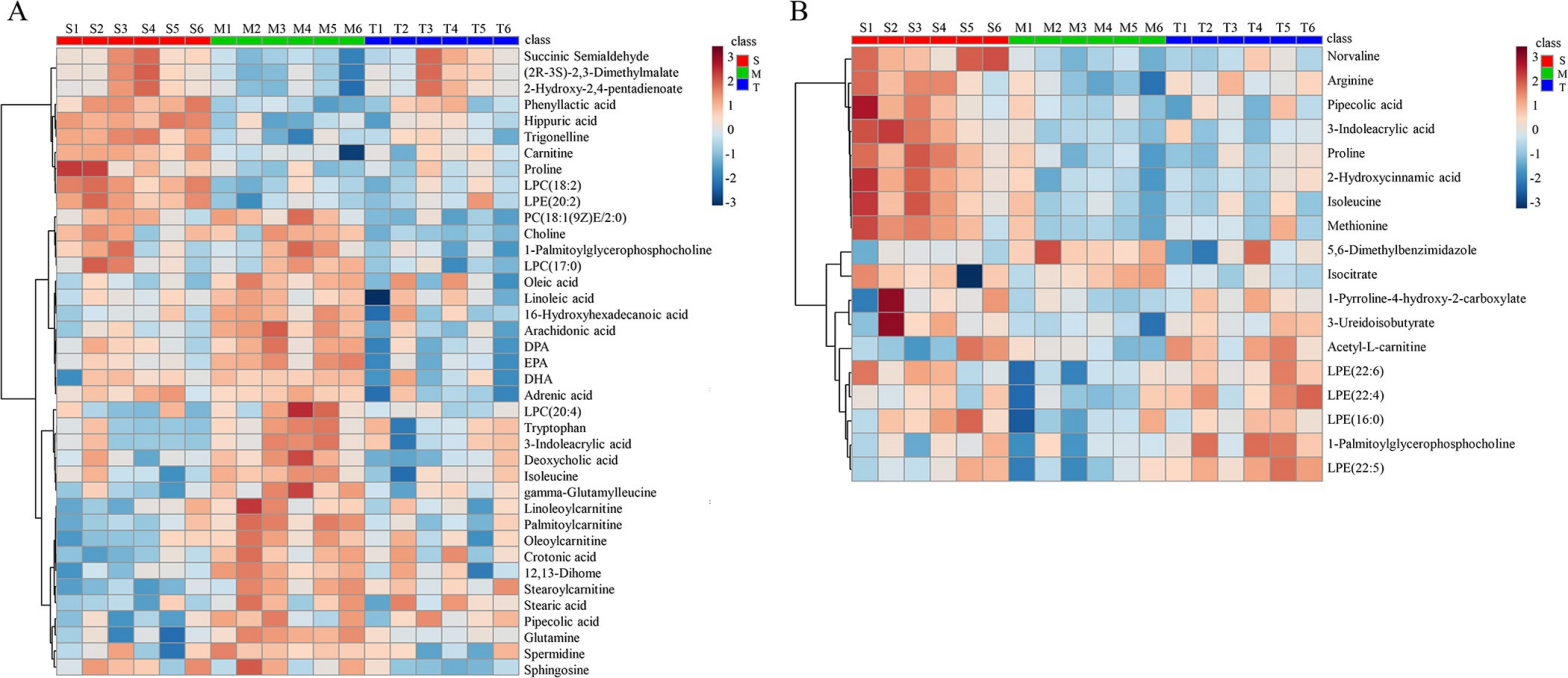
**(A)** The heat map of plasma biomarkers in S, M and T group. **(B)** The heat map of brain biomarkers in S, M and T group.

Heat maps were then applied to reveal the change direction of the brain and plasma metabolites in different groups by MetaboAnalyst 4.0 (shown in **Fig 7A, 7B**). As could be seen from the **Fig 7**, the metabolites of AD rats had changed, and most of the imbalanced metabolites were restored to some extent after administration. As a result, AOF exerted important effects in regulating the metabolic pathways.

### Pathway and correlation network analysis

**Fig 8:**
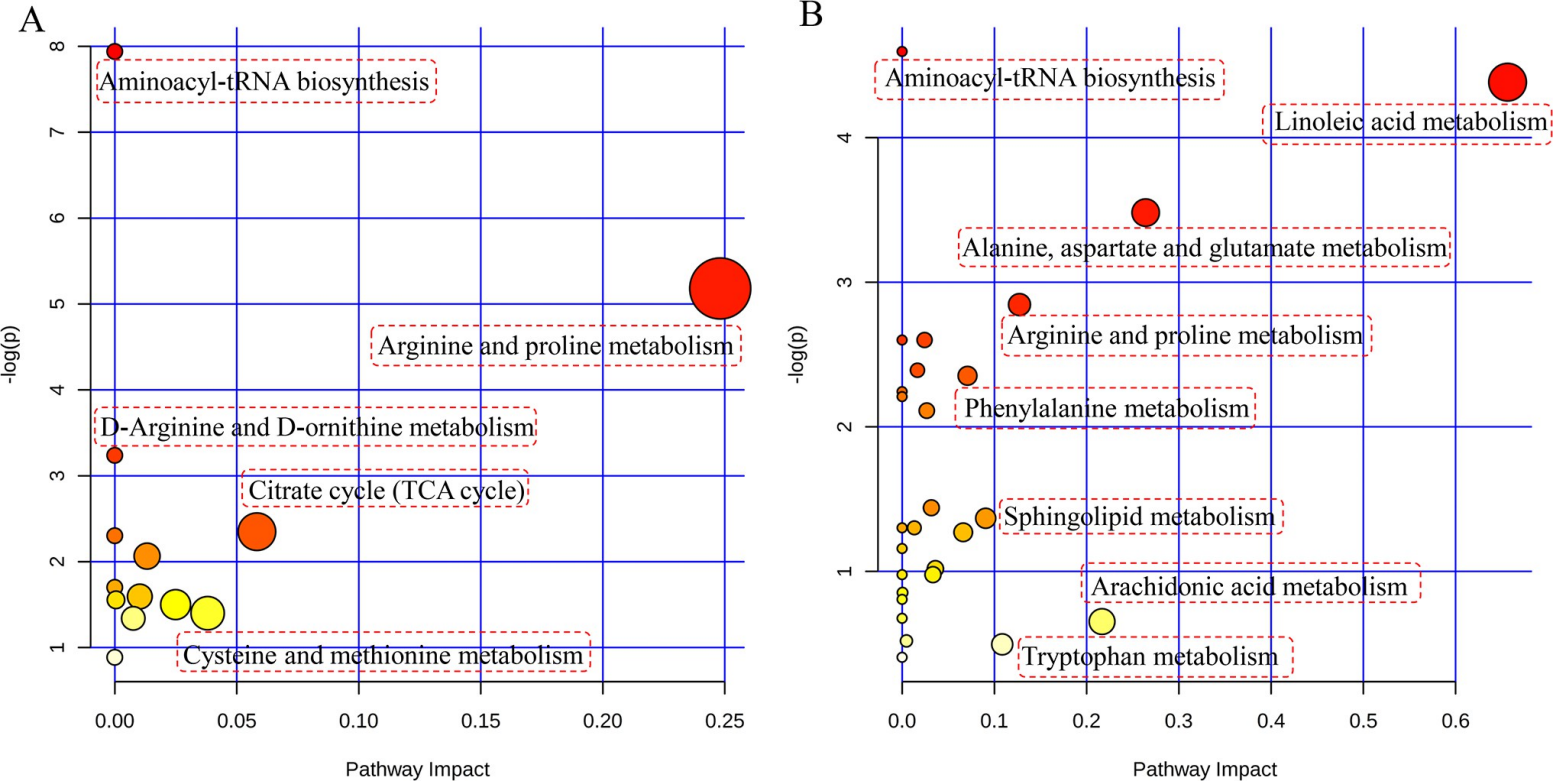
The pathway enrichment analysis of the brain (A) and plasma (B) metabolites in AD regulated by the AOF.

**Fig 9:**
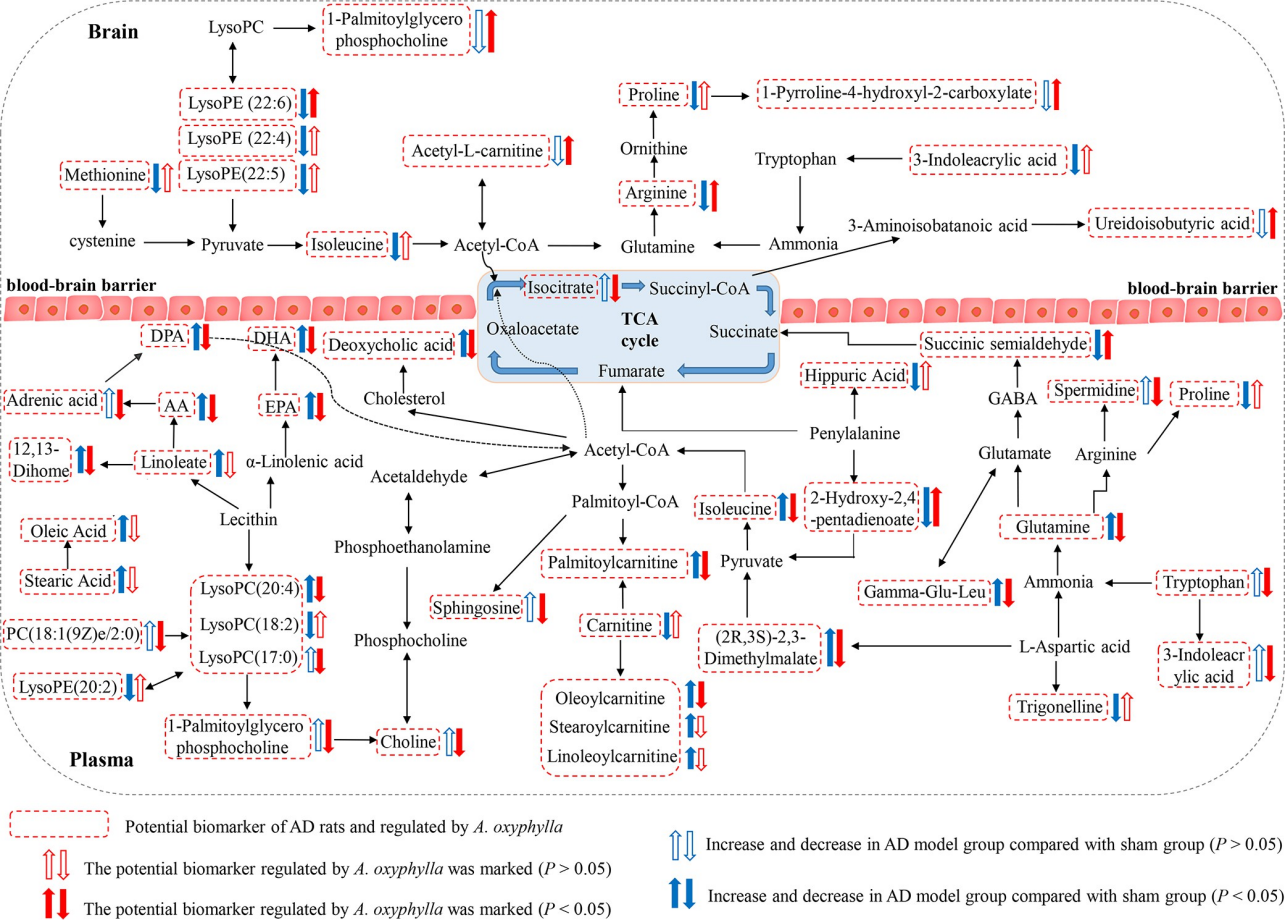
Disturbed metabolic pathway network in AD and the interventional effects of AOF.

To identify the possible metabolic pathways that were affected by AOF on AD model rats, endogenous metabolites in **Tables 1 and 2** were respectively submitted to MetaboAnalyst. Further biological analysis of these pathways had been carried out, and the results showed that the metabolic networks for significantly regulated metabolites in brain were significantly correlated with specific metabolic pathways of arginine and proline metabolism, citrate cycle (TCA cycle) and pyrimidine metabolism, etc **(Fig 8A)**. In plasma, these regulated metabolites were significantly correlated with metabolic pathways of alanine, aspartate and glutamate metabolism, arginine and proline metabolism, sphingolipid metabolism and beta-Alanine metabolism, etc **(Fig 8B)**. Then, a metabolic pathway network linking brain and plasma metabolisms were constructed by searching relevant literature and KEGG database **(Fig 9)**. Metabolites of brain and plasma regulated by.were related to each other via the citrate cycle (TCA cycle).

## Discussion

There are many kinds of chemical constituents in AOF which include essential oils, sesquiterpenes, flavones, diarylheptanoids, glycosides and steroids [16]. the essential oil, the main component of AOF, is highly lipid soluble, easily crosses the blood-brain barrier and is able to fight against neuronal apoptosis, The sesquiterpenes are representative components, such as Nootkatone, which have a clear neuroprotective effect [17]. A significant amount of resources has been designated toward clarifying the protective effect of AOF on AD. However, it remains a big challenge to systematically understand the therapeutic effect of AOF at the metabolic level. We had tried to shed light on this complex regulatory process from the perspective of metabolomics. In this study, 9 brain metabolites and 23 plasma metabolites were adjusted by AOF, which mainly involved amino acid metabolism, lipid metabolism, and energy metabolism. Therefore, we focus on these metabolic pathways and explore how AOF plays its role in the treatment of AD.

### Amino acid metabolism

In the brain, the metabolism of amino acids and their derivatives was disrupted. In our experiment, arginine and 1-pyrroline-4-hydroxy-2-carboxylate of proline derivative showed an obvious downward trend in model group. Arginine level was lower in model group compared to sham group, which was consistent with reports that arginine degradation was associated with AD pathogenesis [18–20], and this effect was reversed by treatment with total extract. The previous study had shown that arginine possesses neuroprotective and anti-apoptotic properties [21]. Besides, arginine is the immediate precursor of nitric oxide (NO), and NO precursor may be effective in reverting cognitive dysfunction associated with AD [22]. As a key semi-essential amino acid, arginine deficiency could lead to cell death. The re-establishment of normal levels of specific amino acids may be a measure of the protective effect of AOF.

In the plasma, the result found that the levels of isoleucine and glutamine in AD rats were higher than in sham group, and 2-hydroxy-2,4-pentadienoate and succinic semialdehyde were lower than in AD rats in the study. The metabolism of amino acids and their derivatives was disrupted as well as brain. In plasma, metabolic disorders of specific amino acids are critical in the progression of AD. For example, essential amino acid isoleucine, a kind of branched-chain amino acid (BCAA), was obtained from diet through food containing protein. BCAAs enter the brain and occur at the blood-brain barrier through competitive transport vectors shared with other large neutral amino acids, including tryptophan and serotonin precursors [23, 24]. Higher plasma BCAA levels could conceivably lead to decreased tryptophan levels in the brain and even had long-term effects on serotonin signaling and downstream pathways, which may increase the risk of AD [23, 25]. Recently, Geng [26] found that, during AD progression, the alteration of gut microbiota composition led to accumulation of plasma isoleucine in the AD mice, which stimulated the differentiation and proliferation of peripheral pro-inflammatory T helper 1 (Th1) cells and promoted their brain infiltration; co-activation of Th1 cells infiltrated into the brain and M1 microglia inherent in the brain led to AD-associated neuroinflammation [27]. Our results showed that the level of amino acids and their derivatives restored back to the control-like levels. After treating with total extract, the metabolic balance of specific amino acids and their derivatives was affected, which provided evidence for the protective effects of AOF on AD.

### Lipid metabolism

The synthesis and degradation of many lipids are considered to be closely related to impairment and repair mechanisms of nerve cells in AD. It is well known that the relational protein of LysoPE is related to the process of neurite growth [28]. In the study, the levels of LPEs decreased in AD model group compared to sham group, which was consistent with previous report of a reduced total LPE concentration [29]. The decreased levels of LPE suggest that cephalin metabolism is compromised in AD rats. LPE is a hydrolysate of phosphatidylethanolamine (PE) under the action of phospholipase A2 (PLA2), a major lipid catabolism enzyme. One study found that the activity of PLA2 was decreased by 35–53% in the parietal and temporal cortices of AD patients; on the contrary, the activities of glycerophosphocholine phosphodiesterase and lysophospholipid acyltransferase, which can promote the synthesis of phospholipids, increased by about 50–70% in AD brain area [30]. These observations may be one of the reasons for the decrease of LPE level in brain tissue of AD rats. Noticeably, increased LPE levels were found in AD rats by treatment with AOF, suggesting that AOF improved AD symptoms by balancing the cephalin metabolism in the brain.

In our study, upregulation of LPC (20:4) had been observed in plasma of AD model rats, and AOF could recover its plasma levels to some extent through oral administration. In addition, the levels of LPC (17:0) and PC(18:1(9Z)e/2:0) were observed downregulated in plasma of AOF treated groups, although their plasma levels in AD model group were not significantly disturbed compared with the sham group. It was reported that up-regulation of LPCs might aggravate the aggregation of β-amyloid protein contribute to increasing apoptotic neuronal death, and inhibition of LPC generation might have a therapeutic effect on AD [31].

The results of this work suggested that unsaturated fatty acids such as arachidonic acid (AA), eicosapentaenoic acid (EPA), (and docosapentaenoic acid(22:5n-6) (DPA), docosahexaenoic acid (DHA) and adrenic acid were increased in AD group. Although the increasing trend of DHA, adrenic acid and DPA was not obvious. The disorder of unsaturated fatty acid metabolism is one of the key factors leading to AD at present. Alpha-linolenic acid (ALA), the precursors of the omega-3 unsaturated fatty acids (DHA, EPA) cannot be synthesized endogenously from carbohydrates and are therefore called essential fatty acids (EFA). DHA and EPA might play a role in cognitive development and memory-related learning in the brain by increasing neural plasticity, synaptic formation and improving synaptic transmission [32]. AA is a common component in mammalian cells [33]. As a biologically active polyunsaturated fatty acid, AA promotes the occurrence and progression of neuroinflammation [34]. It had been reported that neuroinflammation in the hippocampus was a key factor in the progression and onset of AD [35]. the activated microglia by aggregation of Aβ peptide can also induce the synthesis and release of the inflammatory cytokines such as IL-1, and then the activation of the inflammatory cytokines can further increase the expression of AA. The increase in AA levels could increase neuronal activity and mediate Aβ-induced excitotoxicity, leading to learning, memory, and behavioral disorders in AD rat models [36–38]. In the study, the level of plasma AA was higher in AD rats consistent with previous studies [39, 40]. In addition to DHA, EPA and AA, DPA and adrenic acid were detected as potential biomarkers of AD in our study, and all of them were the metabolites of biosynthesis of unsaturated fatty acids metabolism, indicating the influential roles of this metabolism in the pathology of AD. However, it was partly restored by treatment with AOF indicating the therapeutic effect of AOF was related to the regulation of the impaired unsaturated fatty acid metabolism.

### Energy metabolism

The decrease in brain glucose and oxygen metabolism rate is one of the main characteristics of AD. The main pathway for converting glucose to ATP was the TCA cycle. The change of TCA cycle will profoundly change the rate of brain metabolism [41]. It had been reported that many enzymes in TCA cycle of AD patients have obvious damage, resulting in abnormal levels of TCA cycle intermediates and related compounds, such as accumulation of isocitrate in AD rats in our study. After administration, the level of isocitrate increased, which suggested that AOF might play a therapeutic role by regulating the TCA cycle.

Acetyl-l-carnitine contains carnitine and acetyl moiety, both of which have documented neuroprotective effects. Generally, acetyl-l-carnitine as a neuroprotective agent could inhibit the phosphorylation of beta-amyloid precursor proteins, protect neuron cells from lipid peroxidation, regulate memory-related proteins, reduce cell apoptosis and increase the production of nerve growth factors [42–44]. Acetyl-l-carnitine has been proposed to be effective in the treatment of many neurological diseases such as AD and dementia because synaptic energy status and mitochondrial dysfunction are vital factors in their pathogenesis [45]. In our study, although the declining trend of acetyl-l-carnitine in the model group was not obvious, the levels of acetyl-l-carnitine were elevated after administration. Therefore, AOF might alleviate AD though interfering with synthesis of acetyl-l-carnitine.

In the plasma, we observed elevated levels of palmitoylcarnitine and oleoylcarnitine in the model group. Palmitoylcarnitine and oleoylcarnitine, long-chain acyl fatty acid derivative esters of carnitine, are the principal precursors of *β*-oxidation substrate. In the process of biological cells energy metabolism, long-chain acylcarnitines provided fatty acids for β-oxidation through transport from cytoplasm to mitochondria [46, 47]. In this study, the obvious increase of palmitoylcarnitine and oleoylcarnitine in model group may be due to two possibilities: carnitine acyltransferase activity was increased, or beta-oxidation was suppressed. Because carnitine acyltransferase did not change in AD [48], we believed that the main reason for the changes in palmitoylcarnitine and oleoylcarnitine was the inhibition of *β*-oxidation. Previous studies have reported similar results in mouse plasma [49]. The lack of energy aggravated the pathological process of AD Rats. However, their reduction in total extract group indicates that AOF played a therapeutic role by accelerating fatty acid beta-oxidation.

Beyond these pathways mentioned above, many other metabolite disturbances were also adjusted in our study. Some changes were observed in the plasma levels of (2R-3S)-2,3-dimethylmalate and 12,13-Dihome which participate in metabolism of cofactors and vitamins or Linoleic acid metabolism. In addition, the plasma deoxycholic acid in the AD group was obviously higher than sham group, suggesting that secondary bile acid biosynthesis might be associated with AD. AOF might restore the level of these metabolites in the rats of the administration group by intervening in these pathways.

Among the identified differential biomarkers, some biomarkers in brain and plasma were significantly restored after administration (P < 0.05), while the remaining biomarkers were not significantly regulated but had a tendency to be restored (P > 0.05). Among the regulated biomarkers, some of the biomarkers in brain and plasma were obviously imbalanced in AD rats (P < 0.05), while the remaining metabolites were not obviously imbalanced but had a tendency of disorder (P > 0.05). Therefore, AOF played a role in the treatment of AD by regulating the metabolites of clearly or potentially disorder.

## Conclusions

This study could give a preliminary exploration of the significant biologically relevant metabolic changes caused by Aβ-induced AD rats and the underlying mechanisms of AOF According to the experiment results, AOF regulated most of the disordered and potential disordered biomarkers to maintain the balance of the involved pathways. This study indicated that UHPLC-Q-Orbitrap HRMS-based metabolomics could provide a scientific and powerful tool for elucidating the balancing effect of AOF on amino acid, lipid and energy metabolisms in AD rats. In addition, it may be beneficial to better understand TCM’s multi-component and multi-target pharmacological mechanism. Further studies such as quantificational study should be performed for functional validation of significantly altered metabolites to explore the detailed therapeutic mechanism of AOF on AD intervention.

## Supporting information

S1 Fig **Search strategy of rats in the second trial on the second and fifth day.** Traces show the swim path of all groups of rats. S, sham group; M, AD model group; T, AOF group.
(TIF)

S2 Fig The expression levels of Aβ in the hippocampi **(A)** and cerebral cortex **(B)**.Each column represents the mean ± SD for each group (**P < 0.01 versus sham group, ****P < 0.0001 versus sham group; #P < 0.05 versus AD model group, ###P < 0.001 versus AD model group). S, sham group; M, AD model group; T, AOF group.
(TIF)

S3 Fig Representative total ion chromatography (TIC) of brain sample from sham, model and AOF groups in positive mode (A1, B1, C1) and in negative mode (A2, B2, C2). Description of peak position information according to Table 1.
(TIF)

S4 Fig Representative total ion chromatography (TIC) of plasma sample from sham, model and AOF groups in positive mode (A1, B1, C1) and in negative mode (A2, B2, C2). Description of peak position information according to Table 2.
(TIF)

S5 Fig PCA score plot of brain samples and quality controls (QCs) in positive (A) and negative (C) ion mode. PCA score plot of plasma samples and quality controls (QCs) in positive (B) and negative (D) ion mode. S, sham group; M, AD model group; T, AOF group.
(TIF)

S6 Fig The OPLS-DA score plots from M group and T group in positive **(A)** and negative **(C)** ion mode in the brain. The permutations test of M vs. T group in positive **(B)** and negative **(D)** ion mode in the brain. M, AD model group; T, AOF group.
(TIF)

S7 Fig The OPLS-DA score plots from M group and T group in positive (A) and negative (C) ion mode in the plasma. The permutations test of M vs. T group in positive (B) and negative (D) ion mode in the plasma. M, AD model group; T, AOF group.
(TIF)

S1 Table Differential metabolites identified in brain between M group vs. S group. (DOCX)

S2 Table Differential metabolites identified in plasma between M group vs. S group. (DOCX)

S3 Table Regulated metabolites identified in brain between M group vs. T group. (DOCX)

S4 Table Regulated metabolites identified in plasma between M group vs. T group. (DOCX)
