# Supplementary figures and images for: Integrated brain and plasma dual-channel metabolomics to explore the treatment effects of Alpinia oxyphyllaFructus on Alzheimer’s disease
Source: PLoS One. 2023 Aug 8;18(8):e0285401. doi: 10.1371/journal.pone.0285401 (PMC10409282; doi:10.1371/journal.pone.0285401)

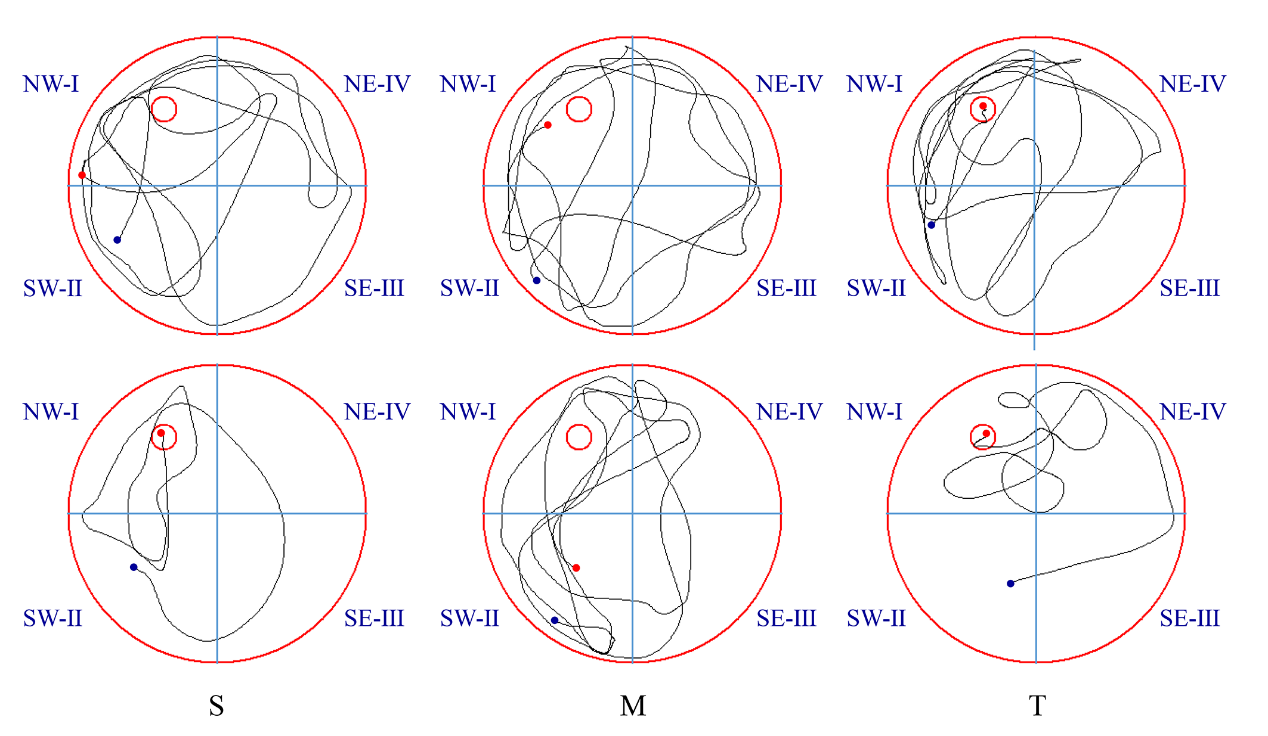

Supplement: S1 Fig — Search strategy of rats in the second trial on the second and fifth day. Traces show the swim path of all groups of rats. S, sham group; M, AD model group; T, AOF group. (TIF) [file pone.0285401.s001.tif]

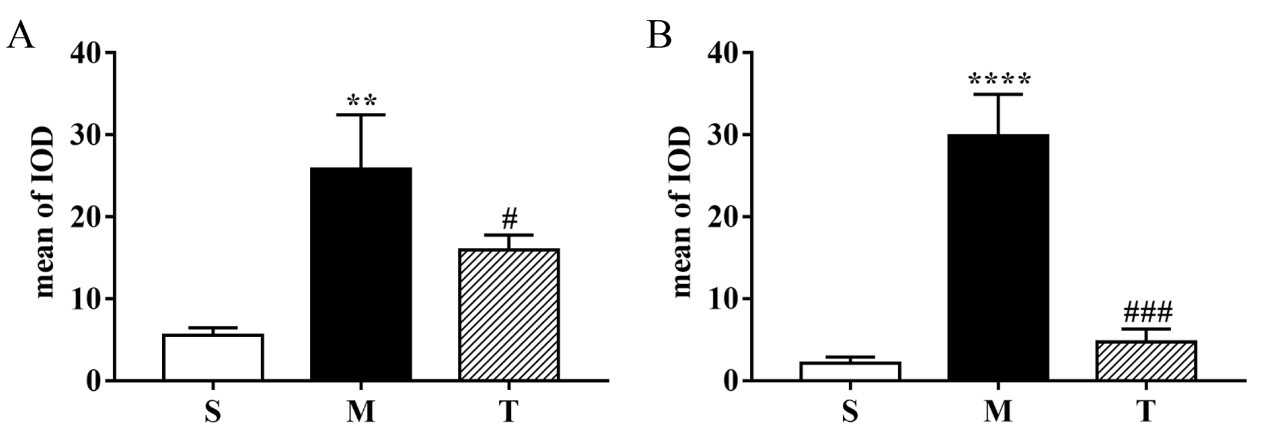

Supplement: S2 Fig — The expression levels of Aβ in the hippocampi (A) and cerebral cortex (B).Each column represents the mean ± SD for each group (**P < 0.01 versus sham group, ****P < 0.0001 versus sham group; #P < 0.05 versus AD model group, ###P < 0.001 versus AD model group). S, sham group; M, AD model group; T, AOF group. (TIF) [file pone.0285401.s002.tif]

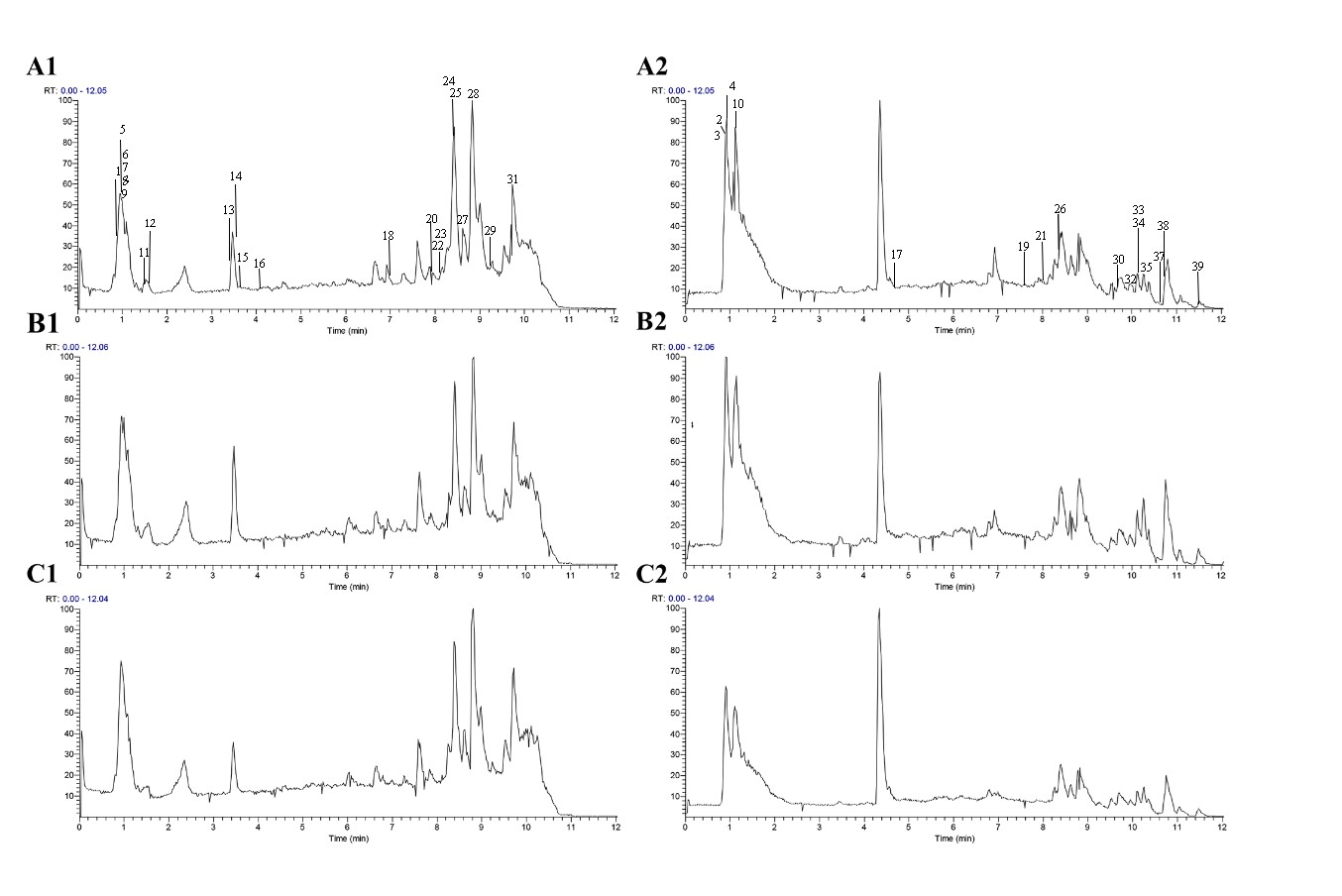

Supplement: S3 Fig — Representative total ion chromatography (TIC) of brain sample from sham, model and AOF groups in positive mode (A1, B1, C1) and in negative mode (A2, B2, C2). Description of peak position information according to Table 1. (TIF) [file pone.0285401.s003.tif]

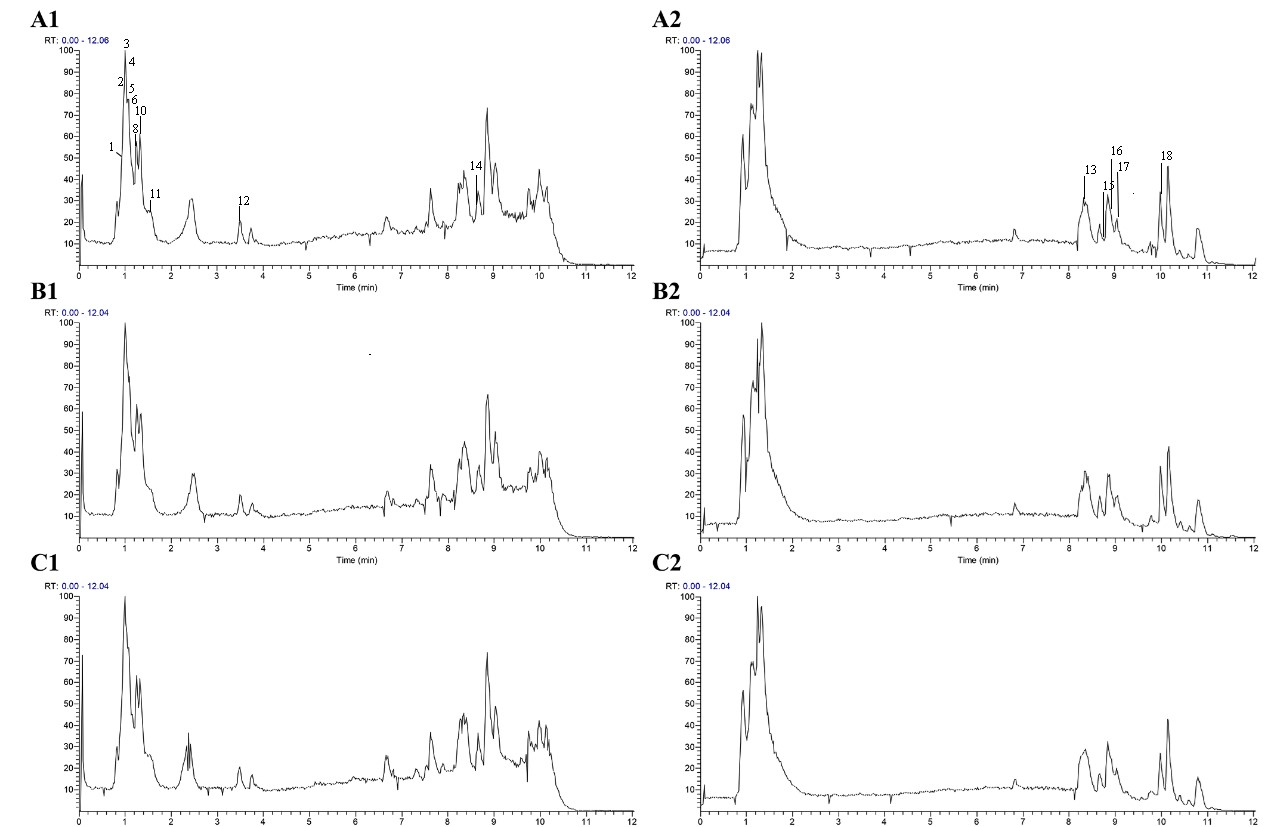

Supplement: S4 Fig — Representative total ion chromatography (TIC) of plasma sample from sham, model and AOF groups in positive mode (A1, B1, C1) and in negative mode (A2, B2, C2). Description of peak position information according to Table 2. (TIF) [file pone.0285401.s004.tif]

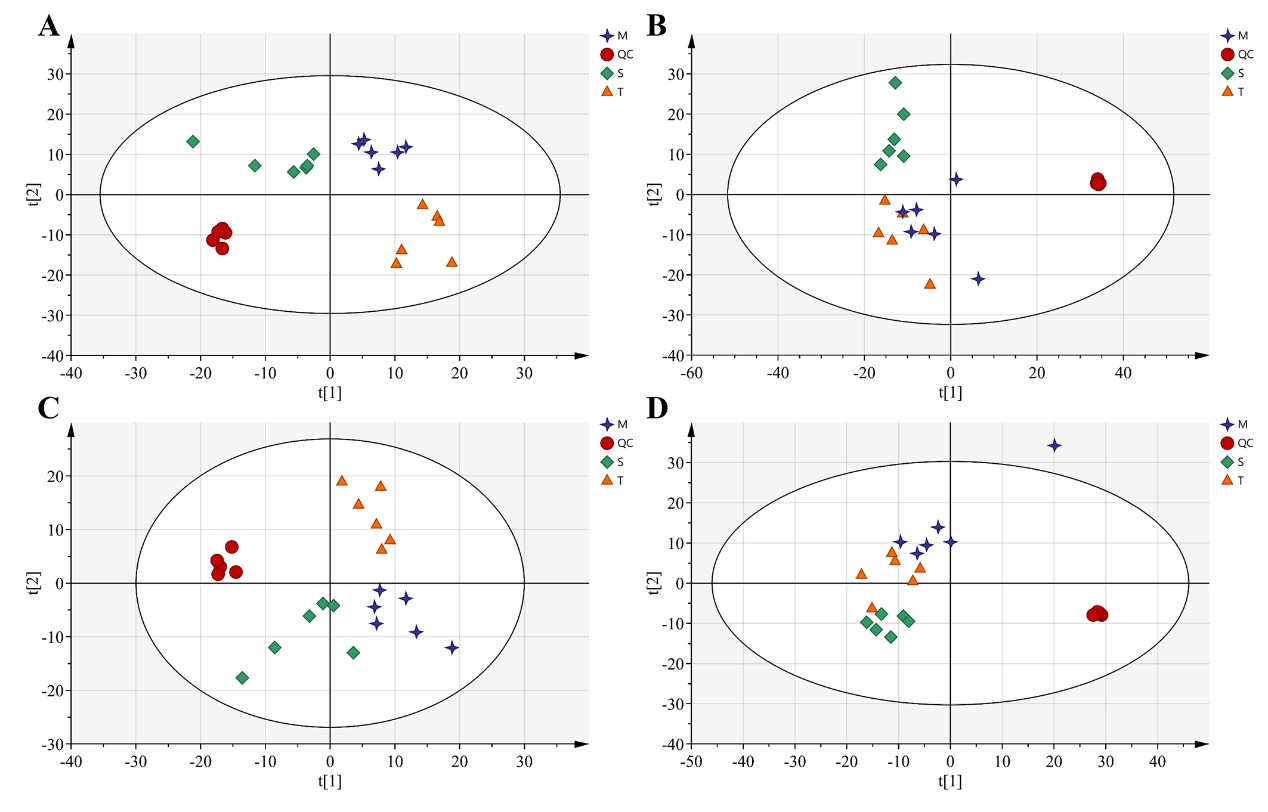

Supplement: S5 Fig — PCA score plot of brain samples and quality controls (QCs) in positive (A) and negative (C) ion mode. PCA score plot of plasma samples and quality controls (QCs) in positive (B) and negative (D) ion mode. S, sham group; M, AD model group; T, AOF group. (TIF) [file pone.0285401.s005.tif]

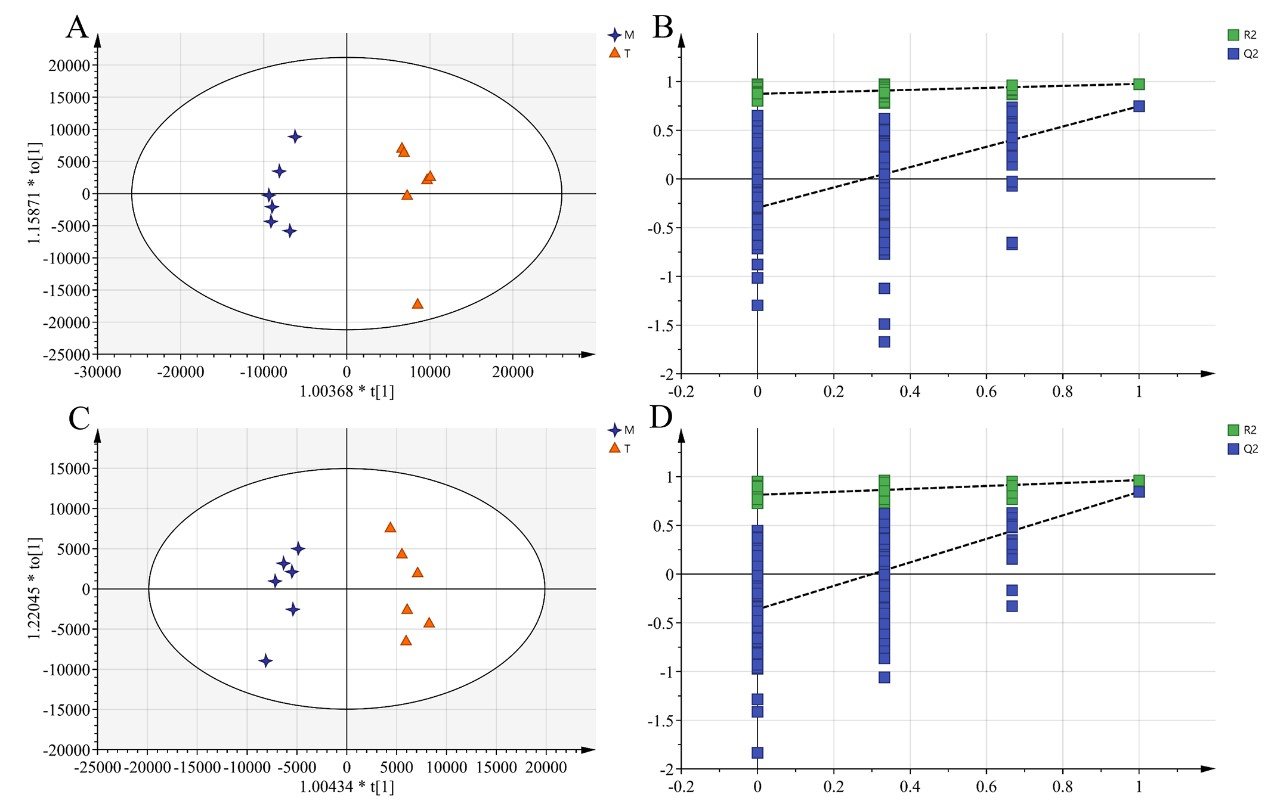

Supplement: S6 Fig — The OPLS-DA score plots from M group and T group in positive (A) and negative (C) ion mode in the brain. The permutations test of M vs. T group in positive (B) and negative (D) ion mode in the brain. M, AD model group; T, AOF group. (TIF) [file pone.0285401.s006.tif]

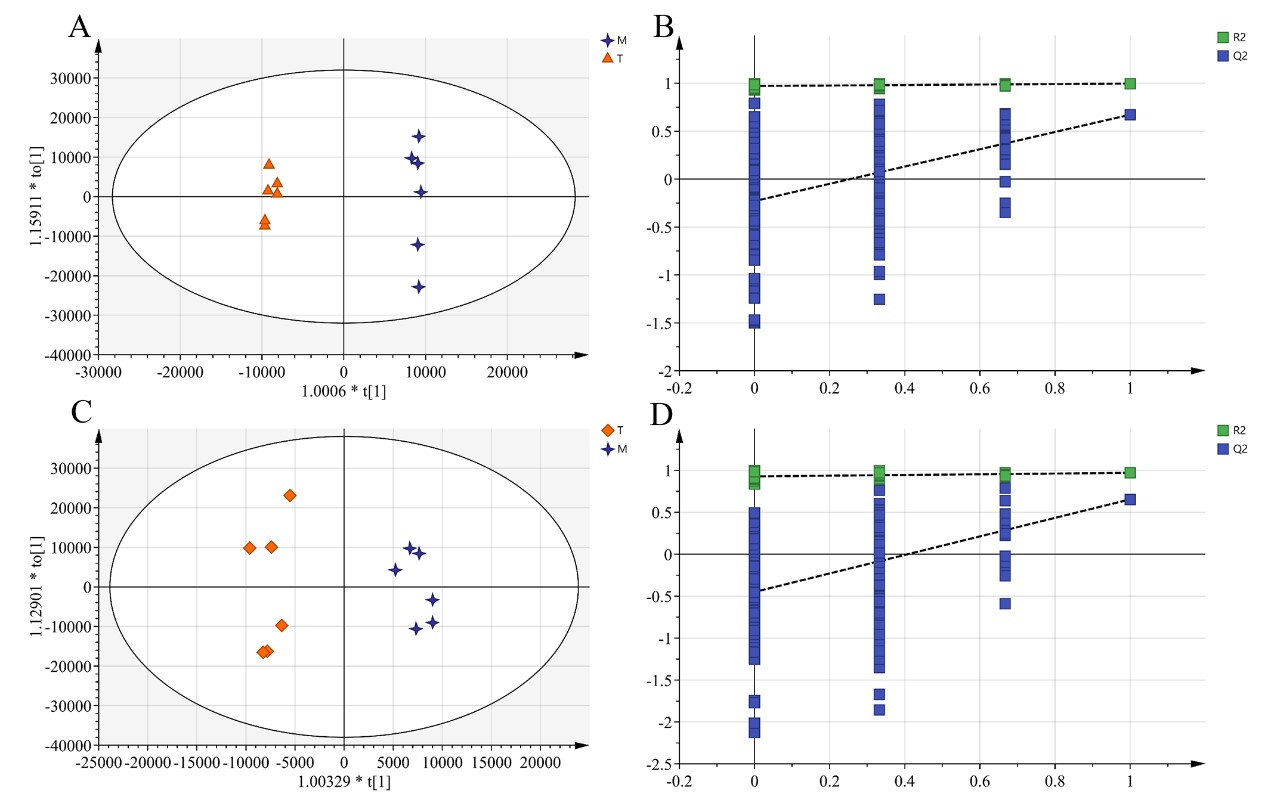

Supplement: S7 Fig — The OPLS-DA score plots from M group and T group in positive (A) and negative (C) ion mode in the plasma. The permutations test of M vs. T group in positive (B) and negative (D) ion mode in the plasma. M, AD model group; T, AOF group. (TIF) [file pone.0285401.s007.tif]
