# Supplementary material for: Integrated brain and plasma dual-channel metabolomics to explore the treatment effects of Alpinia oxyphyllaFructus on Alzheimer’s disease
Source: PLoS One. 2023 Aug 8;18(8):e0285401. doi: 10.1371/journal.pone.0285401 (PMC10409282; doi:10.1371/journal.pone.0285401)
Supplement: S1 Table — (DOCX) [file pone.0285401.s008.docx]

**Table S1.** Differential metabolites identified in brain between M group vs. S group.

| NO | RT (min) | M/Z | Adduct | Metabolites | Formula | Fold change(M/S) | VIP | P-value | MS/MS fragment ion (M/Z) | Δppm |
| --- | --- | --- | --- | --- | --- | --- | --- | --- | --- | --- |
| 1 | 0.84 | 130.04964 | [M+H]+ | Pipecolic acid | C6H11NO2 | 0.768 | 1.526 | 0.019 | 130.05, 112.04, 84.04 | -1.766 |
| 2 | 1.04 | 116.07036 | [M+H]+ | Proline | C5H9NO2 | 0.713 | 3.519 | 0.002 | 116.07, 88.08, 70.07, 56.05 | -2.112 |
| 3 | 1.08 | 118.08600 | [M+H]+ | Norvaline | C5H11NO2 | 0.649 | 2.727 | 0.000 | 118.09, 100.07, 72.08, 55.05 | -2.161 |
| 4 | 1.10 | 175.11896 | [M+H]+ | Arginine | C6H14N4O2 | 0.741 | 1.792 | 0.006 | 175.12, 158.09, 130.10, 116.07, 88.04, 70.07, 60.06 | 0.044 |
| 5 | 1.10 | 150.05803 | [M+H]+ | Methionine | C5H11NO2S | 0.725 | 1.031 | 0.003 | 150.06, 133.03, 104.05, 87.03, 61.01, 56.05 | -1.972 |
| 6 | 1.35 | 165.05428 | [M+H]+ | 2-Hydroxycinnamic Acid | C9H8O3 | 0.717 | 2.638 | 0.002 | 165.05, 147.04, 137.06, 123.04, 119.05, 95.05, 91.05 | -2.064 |
| 7 | 1.58 | 132.10179 | [M+H]+ | Isoleucine | C6H13NO2 | 0.715 | 8.380 | 0.006 | 132.10, 86.10, 69,07 | -0.872 |
| 8 | 3.50 | 188.07016 | [M+H]+ | 3-IndoleacrylicAcid | C11H9NO2 | 0.768 | 5.104 | 0.001 | 188.07, 170.06, 160.08, 146.06, 118.06, 91.05 | -2.367 |
| 9 | 8.34 | 524.27740 | [M-H]- | LysoPE(22:6) | C27H44NO7P | 0.844 | 6.070 | 0.014 | 524.08, 327.23, 214.05, 196.04, 140.01, 78.96 | 0.447 |
| 10 | 8.63 | 496.33804 | [M+H]+ | 1-Palmitoylglycerophosphocholine | C24H50NO7P | 0.852 | 4.547 | 0.011 | 496.34, 478.33, 184.07, 125.00, 104.11, 86.10 | -3.477 |
| 11 | 8.83 | 452.27805 | [M-H]- | LysoPE(16:0) | C21H44NO7P | 0.869 | 6.912 | 0.025 | 452.08, 255.23, 196.04, 140.01, 78.96 | 1.955 |
| 12 | 10.04 | 147.09138 | [M+H]+ | 5,6-Dimethylbenzimidazole | C9H10N2 | 1.391 | 2.671 | 0.004 | 147.10, 132.07, 120.08, 106.06, 79.05, | -2.005 |
