# Supplementary material for: Integrated brain and plasma dual-channel metabolomics to explore the treatment effects of Alpinia oxyphyllaFructus on Alzheimer’s disease
Source: PLoS One. 2023 Aug 8;18(8):e0285401. doi: 10.1371/journal.pone.0285401 (PMC10409282; doi:10.1371/journal.pone.0285401)
Supplement: S2 Table — (DOCX) [file pone.0285401.s009.docx]

**Table S2.** Differential metabolites identified in plasma between M group vs. S group.

| NO | RT (min) | M/Z | Adduct | Metabolites | Formula | Fold change(M/S) | VIP | P-value | MS/MS fragment ion (m/z) | Δppm |
| --- | --- | --- | --- | --- | --- | --- | --- | --- | --- | --- |
| 1 | 0.99 | 161.04559 | [M-H]- | (2R-3S)-2,3-Dimethylmalate | C6H10O5 | 0.664 | 1.994 | 0.006 | 161.05, 131.04, 113.02, 95.01, 85.03, 71.01 | 0.269 |
| 2 | 0.99 | 113.02449 | [M-H]- | 2-Hydroxy-2,4-pentadienoate | C5H6O3 | 0.620 | 1.411 | 0.006 | 113.02, 85.03, 57.03 | 0.643 |
| 3 | 1.00 | 101.02435 | [M-H]- | Succinic Semialdehyde | C4H6O3 | 0.628 | 1.261 | 0.001 | 101.24, 83.01, 73.03, 71.01, 59.01, 55.02 | 10.190 |
| 4 | 1.00 | 116.07023 | [M+H]+ | Proline | C5H9NO2 | 3.385 | 2.939 | 0.007 | 116.07, 88.08, 70.07, 55.05 | 3.232 |
| 5 | 1.03 | 130.08594 | [M+H]+ | Pipecolic Acid | C6H11NO2 | 1.034 | 2.458 | 0.020 | 130.09, 102.05, 84.08, 56.05 | 2.653 |
| 6 | 1.07 | 162.11201 | [M+H]+ | Carnitine | C7H15NO3 | 4.706 | 4.991 | 0.007 | 162.11, 103.04, 85.03, 60.08 | 2.837 |
| 7 | 1.13 | 138.05466 | [M+H]+ | Trigonelline | C7H7NO2 | 0.309 | 2.156 | 0.000 | 138.05, 120.04, 110.06, 94.06, | 2.137 |
| 8 | 1.19 | 145.06180 | [M-H]- | Glutamine | C5H10N2O3 | 1.488 | 1.555 | 0.002 | 145.06, 127.05, 109.04, 101.07, 84.05, 74.02, 58.03 | 7.110 |
| 9 | 1.46 | 87.04388 | [M+H]+ | Crotonic Acid | C4H6O2 | 8.604 | 1.420 | 0.001 | 87.04, 69.03 | 2.022 |
| 10 | 1.54 | 132.10172 | [M+H]+ | Isoleucine | C6H13NO2 | 3.499 | 5.630 | 0.012 | 132.10, 86.10, 69.07 | 1.402 |
| 11 | 3.38 | 205.09705 | [M+H]+ | Tryptophan | C11H12N2O2 | 1.288 | 10.545 | 0.008 | 205.13, 188.07, 170.06, 159.09, 146.06, 132.08, 118.06 | 0.508 |
| 12 | 3.46 | 188.07018 | [M+H]+ | IndoleacrylicAcid | C11H9NO2 | 1.161 | 6.725 | 0.010 | 188.07, 170.06, 146.06, 118.06, 91.05 | 2.260 |
| 13 | 3.63 | 261.14450 | [M+H]+ | Gamma-Glu-Leu | C11H20N2O5 | 1.431 | 1.005 | 0.020 | 261.14, 244.12, 132.10, 86.10 | 0.007 |
| 14 | 4.06 | 180.06494 | [M+H]+ | Hippuric Acid | C9H9NO3 | 1.103 | 1.861 | 0.000 | 180.06, 162.05, 134.06, 105.03, 77.04 | 3.219 |
| 15 | 4.72 | 165.05602 | [M-H]- | Phenyllactic acid | C9H10O3 | 0.223 | 1.178 | 0.000 | 165.06, 147.05, 121.07, 119.05, 73.00 | 8.478 |
| 16 | 7.60 | 313.23871 | [M-H]- | 12,13-Dihome | C18H34O4 | 2.606 | 1.054 | 0.028 | 313.24, 295.23, 277.22, 183.14, 129.09, 99.08 | 4.386 |
| 17 | 7.87 | 424.34094 | [M+H]+ | Linoleoylcarnitine | C25H45NO4 | 0.992 | 1.115 | 0.036 | 424.34, 263.23, 144.10, 85.03, 60.08 | 2.817 |
| 18 | 8.12 | 400.33914 | [M+H]+ | Palmitoylcarnitine | C23H45NO4 | 3.535 | 2.115 | 0.002 | 400.34, 341.27, 239.24, 193.05, 144.10, 129.08, 85.03, 60.08 | 7.482 |
| 19 | 8.19 | 426.35632 | [M+H]+ | Oleoylcarnitine | C25H47NO4 | 1.146 | 1.785 | 0.005 | 426.36, 367.28, 297.21, 265.25, 241.14, 144.10, 85.03, 60.08 | 3.437 |
| 20 | 8.38 | 544.33862 | [M+H]+ | LysoPC(20:4) | C28H50NO7P | 3.187 | 8.236 | 0.049 | 544.33, 526.33, 184.07, 125.00, 104.11, 86.10, 71.07, 60.08 | 2.105 |
| 21 | 8.40 | 520.33862 | [M+H]+ | LysoPC(18:2) | C26H50NO7P | 0.696 | 15.795 | 0.000 | 520.34, 502.33, 337.27, 184.07, 125.00, 104.11, 86.10, 60.08 | 2.202 |
| 22 | 8.41 | 504.30978 | [M-H]- | LysoPE(20:2) | C25H48NO7P | 0.701 | 7.880 | 0.000 | 504.31, 279.23, 224.07, 168.04, 78.96 | 2.606 |
| 23 | 8.55 | 428.37231 | [M+H]+ | Stearoylcarnitine | C25H49NO4 | 0.973 | 1.333 | 0.001 | 428.37, 369.30, 267.27, 144.10, 85.03, | 2.627 |
| 24 | 9.68 | 301.21661 | [M-H]- | Eicosapentaenoic acid | C20H30O2 | 2.049 | 1.825 | 0.003 | 301.22, 257.23, 203.18, 59.01 | 1.339 |
| 25 | 10.11 | 303.23306 | [M-H]- | Arachidonic acid | C20H32O2 | 1.534 | 5.117 | 0.001 | 303.23, 285.22, 259.24, 231.21, 205.20, 177.17, 59.01 | 3.968 |
| 26 | 10.15 | 271.22797 | [M-H]- | 16-Hydroxyhexadecanoic Acid | C16H32O3 | 1.786 | 1.484 | 0.002 | 271.23, 253.22, 225.22, 197.19 | 4.419 |
| 27 | 10.32 | 279.23291 | [M-H]- | Linoleic Acid | C18H32O2 | 1.417 | 4.175 | 0.030 | 279.23, 261.22 | 3.772 |
| 28 | 10.75 | 281.24863 | [M-H]- | Oleic Acid | C18H34O2 | 1.456 | 8.127 | 0.002 | 281.25, 220.14 | 3.994 |
| 29 | 11.48 | 283.26425 | [M-H]- | Stearic Acid | C18H36O2 | 1.631 | 3.167 | 0.025 | 283.26, 265.25, 154.03 | 3.859 |
