# Supplementary material for: Integrated brain and plasma dual-channel metabolomics to explore the treatment effects of Alpinia oxyphyllaFructus on Alzheimer’s disease
Source: PLoS One. 2023 Aug 8;18(8):e0285401. doi: 10.1371/journal.pone.0285401 (PMC10409282; doi:10.1371/journal.pone.0285401)
Supplement: S3 Table — (DOCX) [file pone.0285401.s010.docx]

**Table S3.** Regulated metabolites identified in brain between M group vs. T group.

| NO | RT (min) | M/Z | Adduct | Metabolites | Formula | Fold change(M/T) | VIP | P-value | MS/MS fragment ion (m/z) | Δppm |
| --- | --- | --- | --- | --- | --- | --- | --- | --- | --- | --- |
| 1 | 1.07 | 130.04962 | [M+H]+ | 1-Pyrroline-4-hydroxy-2-carboxylate | C5H7NO3 | 0.831 | 1.649 | 0.018 | 130.05, 102.05, 88.04, 84.04, 74.04, 70.03, 60.04, 56.05 | 1.074 |
| 2 | 1.10 | 175.11896 | [M+H]+ | Arginine | C6H14N4O2 | 0.741 | 1.792 | 0.006 | 175.12, 158.09, 130.10, 116.07, 88.04, 70.07, 60.06 | 0.044 |
| 3 | 1.12 | 145.06136 | [M-H]- | 3-Ureidoisobutyrate | C5H10N2O3 | 0.846 | 1.276 | 0.018 | 145.06, 127.05, 109.04, 101.07, 84.05, 58.03 | 3.485 |
| 4 | 1.23 | 204.12259 | [M+H]+ | Acetyl-L-carnitine | C9H17NO4 | 0.620 | 1.944 | 0.015 | 204.12, 145.05, 85.03 | 2.178 |
| 5 | 1.34 | 191.01935 | [M-H]- | Isocitric Acid | C6H8O7 | 1.316 | 4.961 | 0.013 | 191.02, 173.01, 129.02, 111.01, 85.03 | 1.967 |
| 6 | 8.34 | 524.27740 | [M-H]- | LysoPE(22:6) | C27H44NO7P | 0.844 | 6.070 | 0.014 | 524.08, 327.23, 214.05, 196.04, 140.01, 78.96 | 0.447 |
| 7 | 8.63 | 496.33804 | [M+H]+ | 1-Palmitoylglycerophosphocholine | C24H50NO7P | 0.852 | 4.547 | 0.011 | 496.34, 478.33, 184.07, 125.00, 104.11, 86.10 | 3.477 |
| 8 | 8.79 | 526.29346 | [M-H]- | LysoPE(22:5) | C27H46NO7P | 0.560 | 2.549 | 0.002 | 526.29, 329.25, 196.04, 140.01, 78.96 | 0.860 |
| 9 | 9.05 | 528.30956 | [M-H]- | LysoPE(22:4) | C27H48NO7P | 0.751 | 1.982 | 0.011 | 528.31, 331.26, 196.04, 140.01, 78.96 | 2.664 |
