# Supplementary material for: Integrated brain and plasma dual-channel metabolomics to explore the treatment effects of Alpinia oxyphyllaFructus on Alzheimer’s disease
Source: PLoS One. 2023 Aug 8;18(8):e0285401. doi: 10.1371/journal.pone.0285401 (PMC10409282; doi:10.1371/journal.pone.0285401)
Supplement: S4 Table — (DOCX) [file pone.0285401.s011.docx]

**Table S4.** Regulated metabolites identified in plasma between M group vs. T group.

| NO | RT  (min) | M/Z | Adduct | Metabolites | Formula | Fold change(M/T) | VIP | P-value | MS/MS fragment ion (m/z) | Δppm |
| --- | --- | --- | --- | --- | --- | --- | --- | --- | --- | --- |
| 1 | 0.82 | 146.16475 | [M+H]+ | Spermidine | C7H19N3 | 1.322 | 1.664 | 0.043 | 146.16, 129.14, 112.11, 72.08 | 2.902 |
| 2 | 0.99 | 161.04559 | [M-H]- | (2R-3S)-2,3-Dimethylmalate | C6H10O5 | 0.664 | 1.994 | 0.006 | 161.05, 131.04, 113.02, 95.01, 85.03, 71.01 | 0.269 |
| 3 | 0.99 | 113.02449 | [M-H]- | 2-Hydroxy-2,4-pentadienoate | C5H6O3 | 0.620 | 1.411 | 0.006 | 113.02, 85.03, 57.03 | 0.643 |
| 4 | 1.00 | 101.02435 | [M-H]- | Succinic Semialdehyde | C4H6O3 | 0.628 | 1.261 | 0.001 | 101.24, 83.01, 73.03, 71.01, 59.01, 55.02 | 10.190 |
| 5 | 1.05 | 104.10669 | [M+H]+ | Choline | C5H13NO | 2.009 | 5.689 | 0.001 | 104.11, 60.08 | 2.888 |
| 6 | 1.19 | 145.06180 | [M-H]- | Glutamine | C5H10N2O3 | 1.488 | 1.555 | 0.002 | 145.06, 127.05, 109.04, 101.07, 84.05, 74.02, 58.03 | 7.110 |
| 7 | 1.54 | 132.10172 | [M+H]+ | Isoleucine | C6H13NO2 | 3.499 | 5.630 | 0.012 | 132.10, 86.10, 69.07 | 1.402 |
| 8 | 3.63 | 261.14450 | [M+H]+ | Gamma-Glu-Leu | C11H20N2O5 | 1.431 | 1.005 | 0.020 | 261.14, 244.12, 132.10, 86.10 | 0.007 |
| 9 | 7.46 | 300.29138 | [M+H]+ | Sphingosine | C18H37NO2 | 1.865 | 1.022 | 0.020 | 300.29, 282.28, 264.27, 95.09, 81.07 | 5.575 |
| 10 | 7.60 | 313.23871 | [M-H]- | 12,13-Dihome | C18H34O4 | 2.606 | 1.054 | 0.028 | 313.24, 295.23, 277.22, 183.14, 129.09, 99.08 | 4.386 |
| 11 | 7.98 | 391.28558 | [M-H]- | Deoxycholic acid | C24H40O4 | 3.083 | 1.322 | 0.006 | 391.29, 345.28, 327.27 | 0.504 |
| 12 | 8.12 | 400.33914 | [M+H]+ | Palmitoylcarnitine | C23H45NO4 | 3.535 | 2.115 | 0.002 | 400.34, 341.27, 239.24, 193.05, 144.10, 129.08, 85.03, 60.08 | 7.482 |
| 13 | 8.19 | 426.35632 | [M+H]+ | Oleoylcarnitine | C25H47NO4 | 1.146 | 1.785 | 0.005 | 426.36, 367.28, 297.21, 265.25, 241.14, 144.10, 85.03, 60.08 | 3.437 |
| 14 | 8.38 | 544.33862 | [M+H]+ | LysoPC(20:4) | C28H50NO7P | 3.187 | 8.236 | 0.049 | 544.33, 526.33, 184.07, 125.00, 104.11, 86.10, 71.07, 60.08 | 2.105 |
| 15 | 8.80 | 496.33850 | [M+H]+ | 1-Palmitoylglycerophosphocholine | C24H50NO7P | 1.152 | 12.881 | 0.027 | 496.34, 478.33, 184.07 | 2.550 |
| 16 | 9.25 | 510.35379 | [M+H]+ | LysoPC(17:0) | C25H52NO7P | 1.481 | 4.932 | 0.009 | 515.35, 492.34, 184.07, 104.11, 86.10 | 3.186 |
| 17 | 9.68 | 301.21661 | [M-H]- | Eicosapentaenoic acid | C20H30O2 | 2.049 | 1.825 | 0.003 | 301.22, 257.23, 203.18, 59.01 | 1.339 |
| 18 | 9.79 | 550.38574 | [M+H]+ | PC(18:1(9Z)e/2:0) | C28H56NO7P | 1.726 | 3.122 | 0.001 | 550.39, 532.38, 184.07, 104.11, 86.10 | 1.773 |
| 19 | 9.97 | 327.23306 | [M-H]- | Docosahexaenoic acid | C22H32O2 | 2.179 | 5.286 | 0.002 | 327.23, 283.24, 191.18 | 0.325 |
| 20 | 10.11 | 303.23306 | [M-H]- | Arachidonic acid | C20H32O2 | 1.534 | 5.117 | 0.001 | 303.23, 285.22, 259.24, 231.21, 205.20, 177.17, 59.01 | 3.968 |
| 21 | 10.15 | 271.22797 | [M-H]- | 16-Hydroxyhexadecanoic Acid | C16H32O3 | 1.786 | 1.484 | 0.002 | 271.23, 253.22, 225.22, 197.19 | 4.419 |
| 22 | 10.35 | 329.24881 | [M-H]- | DocosapentaenoicAcid(22n-6) | C22H34O2 | 1.655 | 2.435 | 0.024 | 329.25, 299.24, 285.26, 231.21 | 0.627 |
| 23 | 10.59 | 331.26447 | [M-H]- | Adrenic acid | C22H36O2 | 1.766 | 1.842 | 0.005 | 331.26, 313.25, 287.27, 59.01 | 0.653 |
